# An Adaptive Smoothing-Constrained Broad Learning System for Truck-Scale Weighing

**DOI:** 10.3390/s26185827

**Published:** 2026-09-14

**Authors:** Jing Ling, Jinru Li, Zhimin Wang, Haijun Lin, Dan Xiang

**Affiliations:** 1 School of Artificial Intelligence, Guangzhou Maritime University, Guangzhou 510725, China; danxiang@gzmtu.edu.cn; 2 School of Electronic and Electrical Engineering, Ningxia University, Yinchuan 750021, China; 13356609074@163.com (J.L.); wzm1969851036@163.com (Z.W.); 3 College of Engineering and Design, Hunan Normal University, Changsha 410081, China; linhaijun8010282@126.com

**Keywords:** truck scale, error compensation, broad learning system, maximum correntropy criterion, smoothness constraint

## Abstract

As core weighing equipment in the logistics and industrial sectors, the accuracy of truck scales is significantly affected by environmental noise, sensor errors, and nonlinear factors. This paper proposes an adaptive smoothing-constrained broad learning system (PSO-MCC-SCBLS) to enhance the precision and robustness of truck-scale weighing. Particle swarm optimization (PSO) is employed to optimize the number of feature windows, feature nodes, enhancement nodes, and the smoothing coefficient of the SCBLS; the maximum correntropy criterion (MCC) replaces the minimum mean square error (MMSE) criterion for training the output-weight matrix; and a smoothing constraint derived from the physical continuity of the weighing system is introduced to improve generalization in small-sample scenarios. The method was validated on real data from an 8-channel, 40-ton truck scale under a corrected evaluation protocol that uses group-wise five-fold cross-validation, selects all hyperparameters by an inner cross-validation on the training folds only, and matches the effective regularization strength across MCC and non-MCC variants. A complete component ablation over the seven BLS-family variants (BLS, MCC-BLS, SCBLS, PSO-BLS, PSO-SCBLS, PSO-MCC-BLS and the full model) is reported alongside RBLS, CatBoost, CNN_Attention and LSTM, together with anti-interference tests under a composite Gaussian-plus-impulsive disturbance injected in three scenarios: disturbed calibration only (A), disturbed calibration and deployment (B), and disturbed deployment only (C). The results delimit the contribution of each component. Automated structural search is the one component whose benefit is large and consistent, reducing clean-data RMSE by 47.7% over a plain BLS. On clean data the full model does not lead: PSO-MCC-BLS attains the lowest RMSE ($0.0209\times 10^{3}$ kg) while the full model records $0.0292\times 10^{3}$ kg, indicating that under a leakage-free protocol this calibration task is already close to a low-complexity regime. The advantage of the full model is specific and is reported as such: in Scenario B at a noise ratio of 0.5 it achieves the lowest RMSE ($1.7670\times 10^{3}$ kg) and the best Friedman rank among all eleven models (p<0.05), whereas at the weaker intensity and in Scenario C the deep-learning baselines lead. Once the effective regularization is matched, the MCC term contributes negligibly on this dataset, so the observed robustness is attributable to the tuned BLS-family model as a whole rather than to correntropy weighting in isolation.

## 1. Introduction

With global advancements in logistics and industrial intelligence, truck scales serve as critical equipment for weighing heavy-duty vehicles, playing a pivotal role in sectors such as the commodity trade, traffic regulation, mining, and port transportation. Traditionally, error compensation has primarily relied on technicians manually adjusting potentiometers within individual pressure-sensing channels to calibrate output voltages and offset deviations [1]. Although this approach was once widely implemented, it faces significant limitations. First, the complex signal coupling within load-cell arrays necessitates multiple iterations during the adjustment process, which is both time-consuming and inefficient. Second, due to factors such as eccentric load errors, temperature drift, and nonlinear distortion, manual methods struggle to fully eliminate errors, thereby failing to consistently guarantee weighing accuracy. Furthermore, while classical signal processing techniques, such as Kalman or Butterworth filters, are highly effective at eliminating time-domain dynamic noise, they are inadequate for this specific application. The error compensation in multi-channel truck scales is fundamentally a spatial nonlinear-regression problem rather than a temporal one. Resolving the multi-dimensional spatial nonlinearities caused by static eccentric loading and sensor-specific distortions necessitates the utilization of machine learning models with robust mapping capabilities, rather than purely temporal filtering methods [2].

In recent years, machine learning models have been extensively applied to the field of nonlinear error compensation for sensors. For instance, Liu et al. [3] proposed a weighing-error-compensation method (IGWO-LMBPNN) based on an improved grey wolf optimizer (IGWO) and the Levenberg–Marquardt backpropagation neural network (LMBPNN), which effectively enhanced the weighing precision of truck scales. Similarly, Lin et al. [4] introduced a truck-scale-error-compensation approach based on complex BP neural networks (CBPNNs). This strategy manages errors across various weighing zones by constructing multiple sub-BPNNs and utilizes an adaptive selection network to identify the most suitable sub-BPNN, thereby optimizing compensation performance for different zones.

With the rapid advancement of deep learning and advanced ensemble learning, several cutting-edge data-driven models have been widely adopted for sensor signal processing and regression prediction. For example, long short-term memory (LSTM) networks have become a standard baseline for sensor-array processing due to their exceptional sequence modeling capabilities [5,6]. Convolutional neural networks integrated with spatial attention mechanisms (CNN_Attention) can effectively extract spatial coupling features among multi-channel sensors while dynamically suppressing noise [7]. Furthermore, in tabular regression tasks, advanced gradient-boosting decision tree models, represented by CatBoost, have demonstrated remarkable fitting accuracy [8]. Within the broad learning system (BLS) paradigm, the robust broad learning system (RBLS), which incorporates robust loss functions such as the Huber loss, has also been shown to enhance model noise resistance to a certain extent [9]. However, deep neural networks and complex ensemble models generally suffer from structural complexity, substantial data requirements, and high computational overhead, which limit their deployment efficiency, real-time performance, and generalization capability in industrial scenarios.

The broad learning system (BLS) is an emerging neural network architecture that, by virtue of its unique structure, demonstrates significant advantages in computational efficiency and learning performance when handling high-dimensional data and complex problems. It has been extensively applied across various fields, including image recognition [10,11], fault detection [12,13,14], regression prediction [15,16,17], and time-series forecasting [18,19,20]. Notably, Lin et al. [21] have conducted preliminary research by applying BLS to zero-point tracking in precision electronic balance weighing systems, which initially validated the potential of BLS in nonlinear system modeling and error compensation. However, given the inherent characteristics of truck-scale weighing systems, the direct application of typical BLS algorithms to construct weighing models still encounters several challenges:

(1) Challenges of training-sample scarcity: Data acquisition for truck scales requires the continuous loading of various standard weights at different positions, a process that is both time-consuming and labor-intensive, with high associated costs. In practical operations, it is difficult to obtain large-scale, high-quality training samples that fully cover all potential operating conditions, which severely restricts the model’s generalization capability.
(2) Complexity of structural-parameter optimization: The performance of the BLS is highly dependent on the appropriate configuration of its structural parameters. Traditional tuning methods (such as manual adjustment and grid search) are inefficient in high-dimensional parameter spaces and struggle to guarantee a global optimal solution, often resulting in model accuracy that falls short of the ideal state.
(3) Insufficient robustness in complex environments: The working environments of truck scales are complex and volatile, where factors such as ground vibrations, component aging, and electromagnetic interference introduce significant nonlinear errors. While the standard BLS adopts the minimum mean square error (MMSE) criterion—offering high computational efficiency and fast convergence—it is highly sensitive to outliers and anomalous data points. This sensitivity often leads to degraded robustness when deployed in actual industrial environments.

To address the aforementioned challenges and effectively resolve issues such as the difficulty of data acquisition, the complexity of parameter optimization, and the poor robustness under varying operating conditions in nonlinear modeling for truck scales, this paper proposes an adaptive smoothing-constrained broad learning system based on PSO and MCC (PSO-MCC-SCBLS). The standard BLS is improved and optimized in terms of the following aspects:

Regarding the small-sample learning problem (Challenge 1): Since the incorporation of prior knowledge can effectively reduce the excessive reliance of neural networks on data [1,22], this study applies a mass-change smoothing constraint to improve the network structure of the broad learning system based on weighing theory. The effectiveness of this approach is further analyzed and verified through comparative experiments.

Regarding the complexity of parameter optimization (Challenge 2): To overcome the difficulties in parameter tuning, particle swarm optimization (PSO) is employed for the adaptive optimization of structural parameters [23,24,25]. This study utilizes PSO to perform a global search for key structural parameters of the SCBLS—including the number of feature windows, feature nodes, and enhancement nodes. This approach avoids the traps of local optima inherent in traditional methods, achieving automated and optimal parameter configuration.

Regarding the robustness challenge (Challenge 3): To enhance model robustness, the maximum correntropy criterion (MCC) [26] is introduced to replace the traditional MMSE criterion. By utilizing the high-order statistical features of kernel-weighted errors, MCC maximizes the local similarity between the error signal and the target signal, thereby effectively suppressing the influence of outliers [27,28,29,30]. In this study, the MCC is adopted to optimize the output-weight matrix of the SCBLS, improving the stability and reliability of error compensation.

By integrating global parameter optimization, robustness criteria, and prior-knowledge constraints, this paper constructs a novel error-compensation framework for truck scales capable of simultaneously addressing the issues of small sample sizes, parameter sensitivity, and noise interference. This framework is designed to fully exploit the high-order statistical features of the data, thereby enhancing the system’s adaptability in complex noise environments and providing an effective solution for achieving high-precision and high-robustness weighing. The remainder of this paper is organized as follows: Section 2 elaborates on the theoretical framework and core working principles of the PSO-MCC-SCBLS system; Section 3 focuses on the experimental data-acquisition scheme and the comprehensive evaluation of the system results; finally, Section 4 summarizes the research and suggests directions for future work.

## 2. Truck-Scale Weighing-Error-Compensation Method Based on an Adaptive Smoothing-Constrained Broad Learning System

### 2.1. Weighing Principle of the Truck Scale

**Figure 1:**
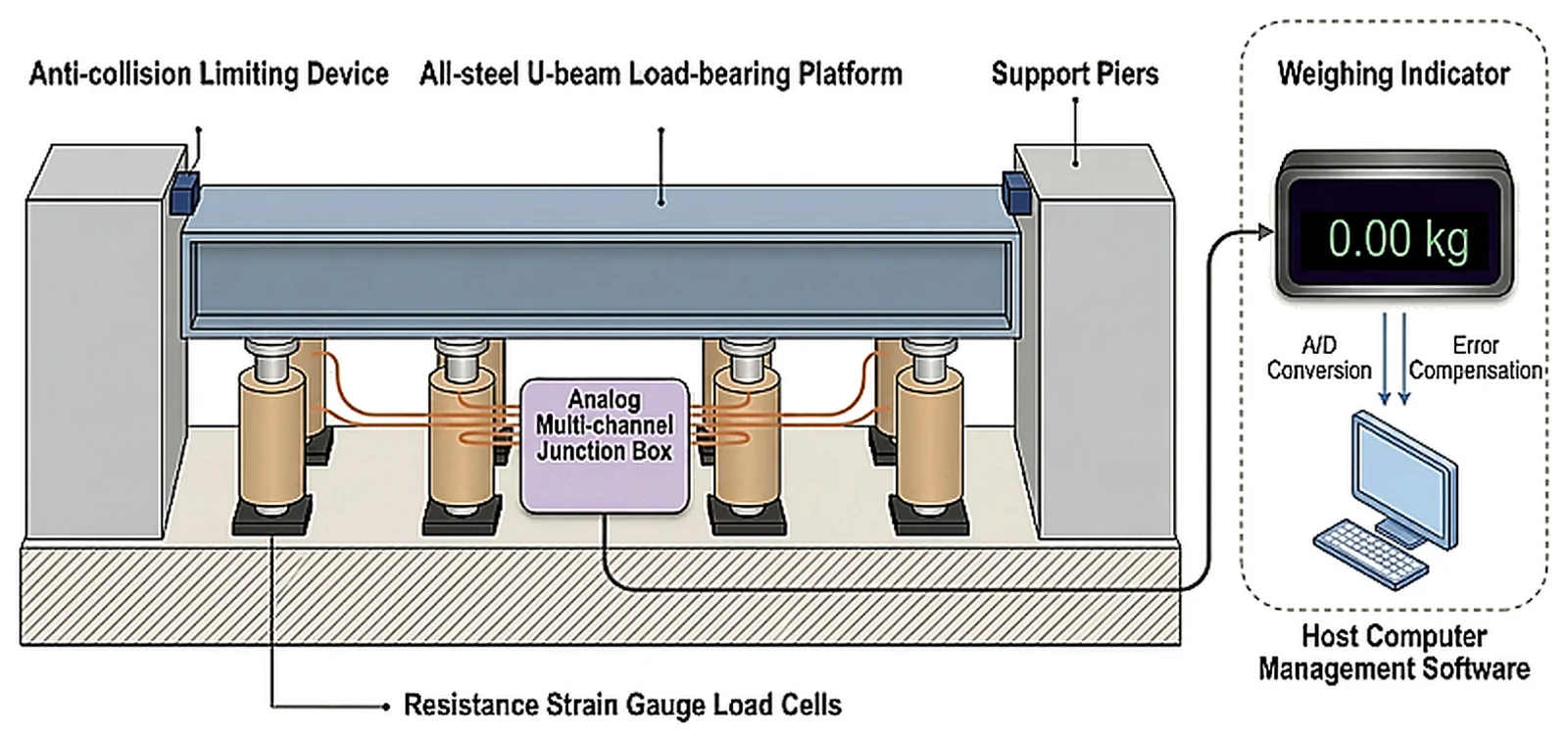
Topological schematic of an 8-channel-sensor electronic truck scale.

Figure 1 illustrates the truck scale and the topological structure of the 8-channel load-cell array. As shown, the truck scale primarily consists of a load-cell array, a scale platform, and a weighing circuit. Under the action of an external force, the elastomer of the load cell undergoes deformation. Consequently, the resistance-strain gauge deforms along with the elastomer, leading to a change in the resistance within the measurement circuit. Ultimately, the relationship between the output voltage $u_{i}$ of the *i*-th load cell and its applied load $x_{i}$ is influenced by multiple physical parameters, exhibiting significant nonlinear characteristics [3]:

(1) $$u_{i}=\frac{K_{i}\frac{g}{S_{i}E_{i}}x_{i}}{1+K_{i}\frac{g}{S_{i}E_{i}}x_{i}\rho_{i}}U_{s}$$

where *g* is the gravitational acceleration; $\delta_{i}$ represents the deformation and strain error of the *i*-th gauge; $S_{i}$ and $E_{i}$ denote the cross-sectional area and Young’s modulus of the *i*-th sensor, respectively; $K_{i}$ is the sensitivity coefficient; and $U_{s}$ represents the input voltage. Incorporating the nonlinear error of the sensors, the weighing result *y* is given by

(2) $$y=\lambda_{p}\left(u_{1}+u_{2}+\cdots +u_{8}\right)=\sum_{i=1}^{8}\lambda_{i}^{p}u_{i}=\sum_{i=1}^{8}\lambda_{i}^{p}\frac{K_{i}Jx_{i}}{1+K_{i}Jx_{i}\rho_{i}}U_{s}$$

where $J=\frac{g}{S_{i}E_{i}}$, and the introduction of strain error $\delta_{i}$ leads to a nonlinear relationship between the weighing result *y* and the applied load $x_{i}$.

The truck-scale system exhibits continuity, where small load variations lead to smooth changes in sensor strain and output voltage, implying a smooth mapping from inputs to weighing results. Accordingly, a data manifold-based smoothness constraint [31] is incorporated into the error-compensation model to enforce similar predictions for similar inputs, which better reflects the system’s physical characteristics than relying solely on training samples.

### 2.2. Weighing Model Based on the Broad Learning System

**Figure 2:**
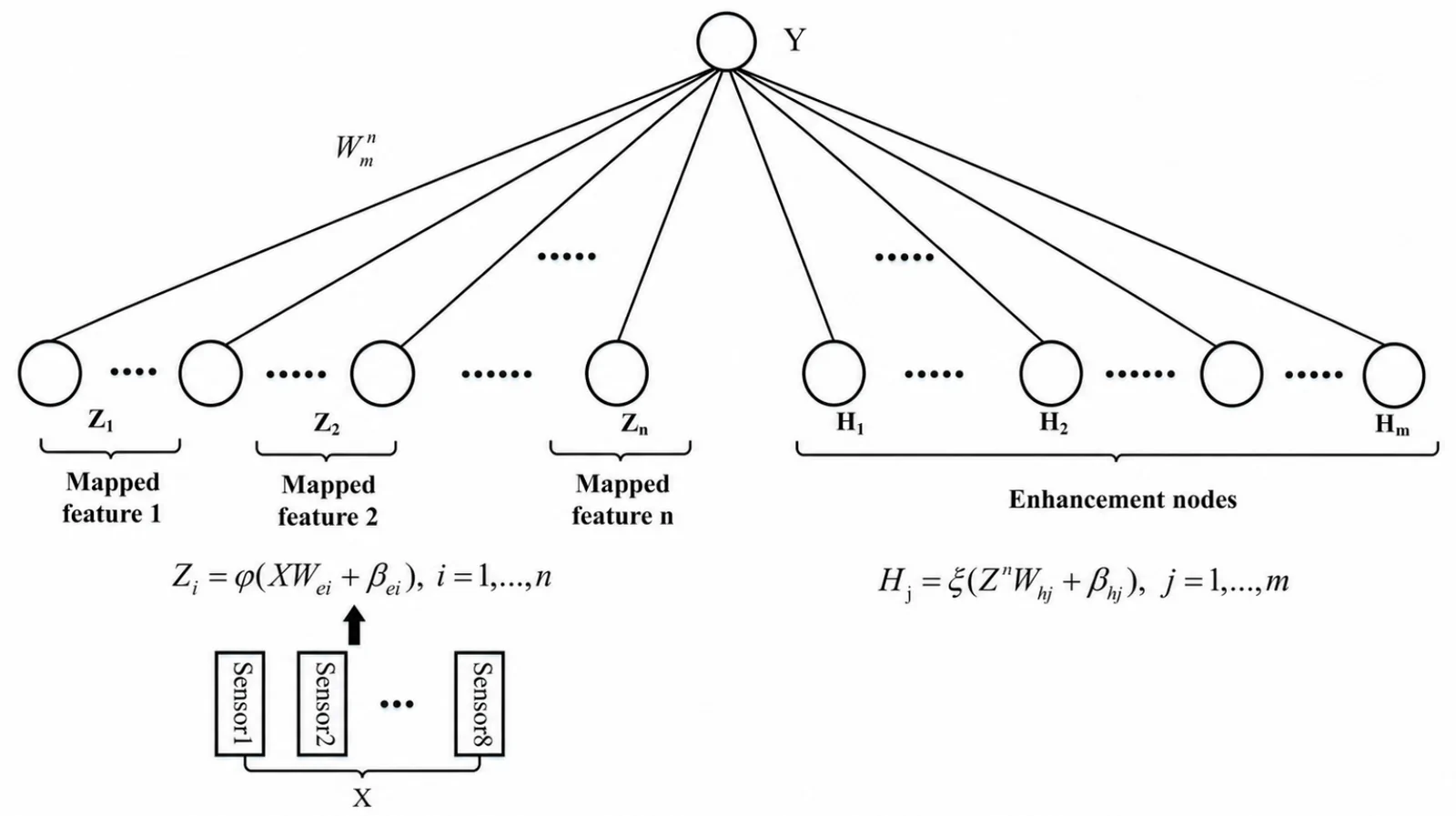
Truck-scale weighing model based on the broad learning system.

As illustrated in Figure 1, the load-cell array is distributed beneath the scale platform according to a specific topology. Outputs are mutually coupled, and the load cells exhibit inherent nonlinearity, making it challenging to construct an accurate error-compensation model for truck scales. The broad learning system (BLS) categorizes hidden layer nodes into feature nodes and enhancement nodes, where the number of nodes can be adjusted based on the system error. This characteristic provides the network with “universal approximation” capabilities and facilitates online updates. By achieving a balance between fitting performance and computational complexity, BLS is highly suitable for the nonlinear modeling of truck scales. The BLS-based truck-scale weighing model is depicted in Figure 2, and comprises an input layer, *n* groups of feature windows (with each group containing *k* feature nodes), *m* enhancement nodes, and an output layer. During the mapping process, the raw data are projected into multiple feature-node groups via random weights. Given a dataset $\left\{\left(X,Y\right)|X\in R^{N\times M},Y\in R^{N\times C}\right\}$, the raw data *X* are first mapped to the feature windows, and the output of the *i*-th feature window is expressed in Equation (3):

(3) $$Z_{i}=\phi \left(XW_{ei}+\beta_{ei}\right),\;i=1,\ldots ,n$$

where ϕ is the linear transformation function for feature mapping, $W_{ei}$ and $\beta_{ei}$ are, respectively, the weight matrix and bias matrix of the feature mapping. During the feature mapping process, the weight matrix and bias matrix are randomly generated.

The outputs of the *n* groups of feature-node windows are combined into $Z^{n}=\left[Z_{1},Z_{2},\ldots ,Z_{n}\right]$, which is then fed into the enhancement-node layer. The output of the *j*-th group of enhancement nodes is calculated as

(4) $$H_{j}=\xi \left(Z^{n}W_{hj}+\beta_{hj}\right),\;j=1,\ldots ,m$$

where ξ is the activation function, $W_{hj}$ and $\beta_{hj}$ are, respectively, the enhancement-mapping weight matrix and bias matrix, both of which are randomly generated during the enhancement-mapping process.

Similarly, the outputs of the *m* enhancement-node groups are combined into $H^{m}=\left[H_{1},H_{2},\ldots ,H_{m}\right]$, and $Z^{n}$ is concatenated with $H^{m}$ to form state matrix *A* [13], where *A* is

(5) $$A=[Z^{n}|H^{m}]$$

The output *Y* is given by

(6) $$Y=[Z^{n}|H^{m}]W=AW$$

where *W* denotes the output-weight matrix. Therefore, obtaining the regularized least-squares solution of *W* becomes the core problem, and the corresponding objective function can be formulated as

(7) $$\arg\min_{W}\left({∥AW-Y∥}_{2}^{2}+\lambda {∥W∥}_{2}^{2}\right)$$

where λ is the ridge regression parameter. During the mapping process, the raw data are projected into multiple groups of feature nodes via random weight matrices. Subsequently, these feature-node groups are mapped to the enhancement nodes through a nonlinear transformation. The feature-node layer and the enhancement-node layer are then concatenated to form the comprehensive feature matrix, from which the output-weight matrix is derived using the ridge regression algorithm. For a detailed description of this model, please refer to reference [32].

### 2.3. Bls Parameter Optimization Based on Particle Swarm Optimization

While the broad learning system (BLS) provides a robust framework for constructing truck-scale weighing models, its performance is highly sensitive to critical structural parameters, including the number of feature windows, feature nodes, enhancement nodes, and the smoothing coefficient. To address this, particle swarm optimization (PSO) is employed in this study to achieve automated and optimal parameter configuration.

Compared with alternative paradigms such as Bayesian optimization (BO)—which is primarily designed for computationally expensive black-box problems under strict evaluation budgets [33]—the choice of PSO is guided by two practical characteristics of SCBLS. First, the closed-form fixed-point solver of SCBLS evaluates in milliseconds, making the surrogate-modeling overhead of BO unnecessary in this setting. Second, PSO directly handles the hybrid search space that combines discrete architectural parameters (*n*, *k* and *m*) with continuous hyperparameters through simple rounding during fitness evaluation, avoiding the surrogate relaxation or acquisition optimization usually needed for mixed-variable BO [33].

PSO is a bio-inspired algorithm that simulates the foraging behavior of bird flocks, identifying the optimal solution through collective cooperation and information exchange among individuals. A population consisting of q particles collaborates to search for the optimum within a D-dimensional space. The mathematical formulation of this process is as follows:

(8) $$v_{i}=\omega v_{i}+c_{1}\mathrm{rand}\left(\right)\left(p_{i}-h_{i}\right)+c_{2}\mathrm{rand}\left(\right)\left(p_{g}-h_{i}\right)$$

(9) $$h_{i}\leftarrow h_{i}+v_{i}$$

where $h_{i}$ and $v_{i}$ denote the position and velocity of the *i*-th particle, $p_{i}$ is its personal best position, and $p_{g}$ is the global best position of the swarm. The inertia weight ω and learning factors $c_{1}$ and $c_{2}$, respectively, govern the particle’s attraction to $p_{i}$ and $p_{g}$. Particle velocities and positions are iteratively updated to search for the optimal solution.

### 2.4. Objective Function of the Broad Learning Network Based on the Maximum Correntropy Criterion

Standard BLS employs the minimum mean square error (MMSE) criterion as the optimization objective when training the output-weight matrix. Intrinsically rooted in the L2-norm, the MMSE criterion squares error terms, which assigns disproportionately high weights to outliers. This often causes the regression curve to be skewed by anomalous points, thereby compromising the fitting accuracy for normal samples.

In contrast, the maximum correntropy criterion (MCC) maps data into a high-dimensional feature space via kernel functions, utilizing local similarity to quantify error. Regarding transient impacts or large-amplitude anomalous fluctuations common in truck-scale signals, MCC leverages the inherent properties of the Gaussian kernel function. Specifically, as the error magnitude increases, the corresponding kernel-mapping value rapidly decays toward zero. This mechanism enables MCC to automatically suppress large errors by assigning minimal weights to outliers, allowing the system to focus primarily on normal sample regions characterized by smaller errors. Consequently, MCC serves as a more suitable and robust alternative for operation in noisy environments.

Correntropy [34], as a generalized correlation function, serves as a metric for characterizing the similarity between feature vectors. For any two random variables X and Y, the correntropy can be defined as follows:

(10) V(X,Y)=E[k(X,Y)]

where E[·] denotes the expectation function after the Gaussian transformation of the difference between the two feature vectors *X* and *Y*, and k(·) represents a kernel function that satisfies Mercer’s condition. In this study, the Gaussian kernel function [35] is adopted by default, as shown in Equation (11):

(11) $$k_{\sigma }\left(x_{i},y_{i}\right)=\frac{1}{\sqrt{2\pi }\sigma }\exp\left(-\frac{∥x_{i}-y_{i}{∥}_{2}^{2}}{2\sigma^{2}}\right)$$

where σ represents the kernel width, which is utilized to control the weight of higher-order statistics. However, in most practical scenarios, the joint-probability density function of the feature vectors *X* and *Y* is unknown. Therefore, the sample estimator is used as an approximation for the expectation function in Equation (10). By incorporating the Gaussian kernel function, it yields

(12) $$V\left(X,Y\right)=\frac{1}{N}\sum_{i=1}^{N}k_{\sigma }\left(x_{i},y_{i}\right)$$

Ultimately, MCC optimizes model parameters by maximizing the correntropy, with the objective function defined as follows:

(13) $$J\left(\theta \right)=\frac{1}{N}\max_{\theta }\sum_{i=1}^{N}k_{\sigma }\left(x_{i},y_{i}\right)$$

By integrating MCC into the BLS framework, the modified optimization objective function is obtained, as shown in Equation (14):

(14) $$J\left(W\right)=\arg\max_{W}\left(\sum_{i=1}^{N}\exp\left(-\frac{∥a_{i}W-y_{i}{∥}_{2}^{2}}{2\sigma^{2}}\right)-\frac{\lambda }{2}{∥W∥}_{2}^{2}\right)$$

where $a_{i}$ denotes the *i*-th row of the state matrix *A*, $y_{i}$ represents the corresponding target output, and *W* is the output-weight matrix to be optimized.

To enhance the model’s generalization ability under small-sample conditions and noisy environments, this study introduces a graph Laplacian matrix to construct a manifold regularization term. The underlying assumption is that if two samples are adjacent in the feature space, their predicted outputs should also be similar. To this end, an adjacency matrix $S\in R^{N\times N}$ is first constructed to measure the similarity between sample *i* and sample *j*. The similarity weight $S_{ij}$ is defined using a Gaussian kernel function, which can be expressed as

(15) $$S_{ij}=\left\{\begin{array}{cc} \exp\left(-\frac{∥x_{i}-x_{j}{∥}_{2}^{2}}{2\sigma_{s}^{2}}\right), & i\neq j,\\ 0, & i=j, \end{array}\right.$$

where $\sigma_{s}$ denotes the kernel width parameter. The degree matrix *D* is defined as a diagonal matrix whose diagonal elements are $D_{i}=\sum_{j=1}^{N}S_{ij}$. Accordingly, the graph Laplacian matrix *L* is defined as

(16) L=D−S

Therefore, the manifold regularization term can be expressed as

(17) $$R_{\mathrm{smooth}}=\frac{1}{2}\sum_{i=1}^{N}\sum_{j=1}^{N}S_{ij}{\left(y_{i}-y_{j}\right)}^{2}=\mathrm{Tr}\left(Y^{T}LY\right)$$

This term penalizes large output discrepancies between similar samples through the Laplacian matrix *L*, thereby enforcing a smoothness constraint that preserves the local structure of the data. By incorporating the manifold regularization term into the objective function in Equation (14), the updated objective function is given by

(18) $$J\left(W\right)=\arg\max_{W}\left(\sum_{i=1}^{N}\exp\left(-\frac{∥a_{i}W-y_{i}{∥}_{2}^{2}}{2\sigma^{2}}\right)-\frac{\lambda }{2}{∥W∥}_{2}^{2}-\frac{\lambda_{\mathrm{smooth}}}{2}\mathrm{Tr}\left(W^{T}A^{T}LAW\right)\right)$$

where $\lambda_{\mathrm{smooth}}$ is the manifold regularization coefficient. Taking the derivative of the objective function with respect to the output-weight matrix *W* and setting the gradient to zero, the gradient contributed by the manifold regularization term is $\frac{\partial \mathrm{Tr}(W^{T}A^{T}LAW)}{\partial W}=2A^{T}LAW$. The resulting gradient expression is given by

(19) $$\frac{\partial J(W)}{\partial W}=-\frac{1}{\sigma^{2}}\sum_{i=1}^{N}a_{i}^{T}\exp\left(-\frac{∥a_{i}W-y_{i}{∥}_{2}^{2}}{2\sigma^{2}}\right)\left(a_{i}W-y_{i}\right)-\lambda W-\lambda_{\mathrm{smooth}}A^{T}LAW$$

The matrix form of Equation (19) can be written as follows:

(20) $$\frac{\partial J(W)}{\partial W}=-\frac{1}{\sigma^{2}}A^{T}\Lambda_{w}\left(AW-Y\right)-\lambda W-\lambda_{\mathrm{smooth}}A^{T}LAW$$

where $\Lambda_{w}$ is the diagonal element of the output-weight matrix, calculated as shown in Equation (21), with each element obtained by applying the Gaussian kernel function to the corresponding sample error. The update formula is given by

(21) $$\Lambda_{w}=\mathrm{diag}\left[\exp\left(-\frac{∥a_{1}W-y_{1}{∥}_{2}^{2}}{2\sigma^{2}}\right),\ldots ,\exp\left(-\frac{∥a_{N}W-y_{N}{∥}_{2}^{2}}{2\sigma^{2}}\right)\right]$$

(22) $$W={\left[A^{T}\Lambda_{w}A+\gamma I+\lambda_{\mathrm{smooth}}\sigma^{2}A^{T}LA\right]}^{-1}A^{T}\Lambda_{w}Y$$

where $\gamma =\lambda \sigma^{2}$, and since $\Lambda_{w}$ depends on the output-weight matrix *W*, the fixed-point iteration method is employed. With the initial value set as $W_{0}$, the weight-update rule in each iteration can be expressed as

(23) $$W_{t+1}={\left[A^{T}\Lambda_{w}\left(W_{t}\right)A+\gamma I+\lambda_{\mathrm{smooth}}\sigma^{2}A^{T}LA\right]}^{-1}A^{T}\Lambda_{w}\left(W_{t}\right)Y$$

The iteration-stopping condition is $∥W_{t+1}-W_{t}{∥}_{2}<\varepsilon$, where ε is the termination tolerance. When the updated value of the output-weight matrix converges within a certain range, the iteration stops, and the optimal output-weight matrix is finally output for the SCBLS error-compensation model.

The pseudocode for the truck-scale weighing model based on PSO-MCC-SCBLS is presented in Algorithm 1.
**Algorithm 1** The PSO-MCC-SCBLS Algorithm for Truck-Scale Weighing**Input:** Training dataset of truck-scale weighing data.**Output:** Predicted output *Y* for the test dataset.  1:Data preprocessing.  2:for i=1; i≤n do.  3:Randomly initialize the parameters $W_{e}$ and $\beta_{e}$ of the feature mapping layer;  4:Calculate $Z_{i}=\phi \left(XW_{ei}+\beta_{ei}\right),\;i=1,\ldots ,n$;  5:end  6:Construct feature-node matrix $Z^{n}=\left[Z_{1},Z_{2},\ldots ,Z_{n}\right]$;  7:for j=1; j≤m do  8:Randomly initialize the parameters $W_{h}$ and $\beta_{h}$ of the enhancement-mapping layer  9:Calculate $H_{j}=\xi \left(Z^{n}W_{hj}+\beta_{hj}\right),\;j=1,\ldots ,m$;10:end11:Construct enhancement-node matrix $H^{m}=\left[H_{1},H_{2},\ldots ,H_{m}\right]$;12:Concatenate node matrices to form $A=[Z^{n}|H^{m}]$;13:Compute the adjacency matrix *S* and the Laplacian matrix *L* using Equations (15) and (16);14:for $t_{1}=1$; $t_{1}\leq T_{1}$ do15:Initialize output-weight matrix *W* and construct state matrix *A* based on Equations (3)–(6);16:Compute diagonal matrix $\Lambda_{w}$ according to Equation (21);17:Update output-weight matrix *W* according to Equation (22);18:if $∥W_{t+1}-W_{t}{∥}_{2}\;<\varepsilon$;19:break20:end21:Output-weight matrix *W*;22:for $t_{2}=1$; $t_{2}\leq T_{2}$ do23:Evaluate fitness of each particle;24:Update personal best and global best positions;25:Update particle velocity and position using Equations (8) and (9);26:end27:Output the optimized structural parameters of SCBLS;28:Train the model using the optimal parameters;29:Output *Y*;30:return *Y*

## 3. Experimental Studies

### 3.1. Weighing-Data Collection

**Figure 3:**
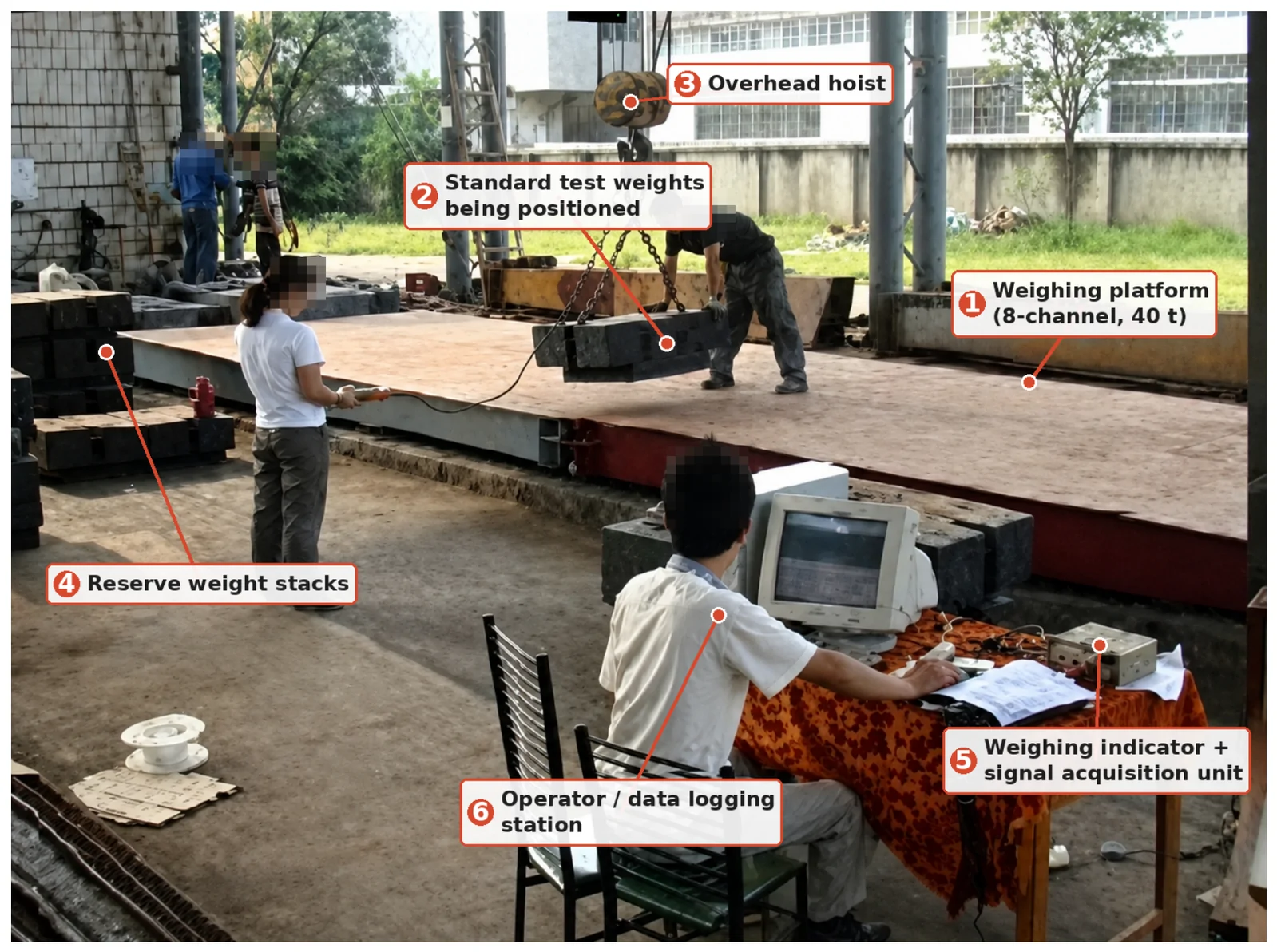
Physical experimental setup for truck-scale weighing-data collection: (1) weighing platform (8-channel, 40 t); (2) standard test weights being positioned; (3) overhead hoist; (4) reserve-weight stacks; (5) weighing indicator and signal-acquisition unit; and (6) operator/data-logging station.

Figure 3 shows the physical experimental setup used to collect the calibration data.

The truck scale used in this study consists of eight 20-ton load cells with a maximum capacity of 40 tons, 4000 divisions, and a scale interval of 10 kg. Standard weights of 0.5, 1, 6, and 10 tons are repeatedly applied at different positions, yielding 162 samples collected via the weighing-signal-acquisition circuit. Each sample comprises the eight load-cell outputs $X_{i}$ and the corresponding target weight $Y_{i}$.

Due to the time-consuming and costly on-site calibration process, the dataset is relatively limited. The 162 samples, in fact, form 81 groups of two repeated measurements taken at the same nominal load and loading position; within a group, the two measurements are near-duplicates of each other (the median within-group Euclidean distance is more than two orders of magnitude smaller than the distance to the next-nearest sample). Accordingly, this work aims to develop a model with strong generalization under small-sample conditions by incorporating a smoothness-constrained physical prior into the BLS framework and employing five-fold cross-validation. After preprocessing and normalization, the 162 samples are divided into five folds *by group* (GroupKFold), so that both replicates of a group always fall on the same side of the split. A random, non-group-aware split was found to place the two replicates of the same group on opposite sides of the train/test boundary for 80.2% of the test samples, which allows a model to recognize a near-duplicate of a training point instead of generalizing to it; every cross-validation result reported in this paper, therefore, uses the group-wise split. The model is trained on four folds and tested on the remaining one, repeated five times so that each fold serves once as the test set. Final performance is reported as the mean and standard deviation over all folds.

### 3.2. Parameter Settings of the PSO-MCC-SCBLS System

The SCBLS system employs the tansig function as its activation function. The ridge regression parameter is not fixed but is one of the quantities optimized by PSO (see below); the value $2^{-10}$ is used only to initialize the search. For the MCC weight update, the maximum number of iterations is 10 and the fixed-point iteration terminates when $∥W_{t+1}-W_{t}{∥}_{2}\;<\varepsilon$ with $\varepsilon =10^{-3}$. The PSO-MCC-SCBLS system utilizes RMSE, MAE, and MAPE as evaluation metrics, calculated as shown in Equations (24)–(26). Here, $y_{i}$ denotes the true value, ${\hat{y}}_{i}$ denotes the predicted value, and *n* is the number of samples.

To optimize the PSO-MCC-SCBLS system, a parameter-tuning procedure is conducted using PSO with 10 particles in a seven-dimensional search space. The inertia weight, cognitive coefficient, and social coefficient are set to 0.8, 1.9, and 1.5, respectively. Each particle encodes a parameter set $\left(k,n,m,\log_{2}\gamma ,\lambda_{\mathrm{smooth}},\sigma_{s},\log_{2}\sigma \right)$, where the number of feature nodes per window *k*, the number of feature windows *n* and the number of enhancement nodes *m* are discrete integers and particle positions are rounded during fitness evaluation; $\sigma_{s}$ is the kernel width of the smoothness-graph adjacency matrix of Equation (15), σ is the MCC kernel width, and γ is the effective ridge penalty. Two nested bound settings are used. The clean-data comparison of Section 3.3 and Section 3.4 uses an extended space, k∈[2,50], n∈[1,10], m∈[2,500], $\log_{2}\gamma \in \left[-30,-2\right]$, $\lambda_{\mathrm{smooth}}\in \left[10^{-9},0.2\right]$, $\sigma_{s}\in \left[0.02,8.0\right]$ and $\log_{2}\sigma \in \left[-8,8\right]$, which is wide enough to let the search reduce the network towards a near-linear model if the data call for it. The anti-interference experiments of Section 3.5 use the narrower six-dimensional space k∈[10,50], n∈[1,10], m∈[100,500], $\log_{2}\gamma \in \left[-14,-2\right]$, $\lambda_{\mathrm{smooth}}\in \left[10^{-6},5\times 10^{-2}\right]$ and $\sigma_{s}\in \left[0.2,3.0\right]$ with the MCC kernel width held fixed at $\sigma =2^{5}$, since the noise matrix requires an independent search for every (scenario, α, fold, and noise realization) cell and a smaller space keeps that affordable. The setting used is stated with each table. Variants without the PSO- prefix are tuned by a random grid search over the same bounds and with the same number of fitness evaluations, so that the ablation compares search strategies at an equal tuning budget. Because the MCC objective in Equation (22) enters the normal equations through $\gamma =\lambda \sigma^{2}$ rather than through λ itself, searching a common interval for the ridge parameter λ would give the MCC and the non-MCC variants effective ridge penalties differing by a factor of $\sigma^{2}$. All results reported below, therefore, search $\log_{2}\gamma$ directly and recover $\lambda =\gamma /\sigma^{2}$ for the MCC variants, so that every model is compared at the same effective regularization strength.

Hyper-parameters are selected by an inner 3-fold cross-validation computed on the training folds only; the outer test fold is never used for model selection. In each inner-CV evaluation, the SCBLS model is built using the current particle’s parameters, the output weights are computed via the MCC criterion, and the mean validation-fold RMSE serves as the PSO fitness function. Particle positions exceeding the specified bounds are truncated. After 50 iterations, the particle swarm’s global-best parameters are obtained and the model is refitted on the full training fold for testing. Because the search is repeated independently on every training fold, the selected structure varies from fold to fold rather than being a single global setting; across the five folds and 30 repetitions the median selected values are n=1 feature window, k=9 feature nodes per window and m=2 enhancement nodes, with the number of enhancement nodes driven to the lower bound of the search interval in every fold. Each of the 30 repetitions uses an independently seeded random-number stream governing both the random feature/enhancement mappings and the PSO search itself, so the reported standard deviation reflects genuine repetition-to-repetition variability rather than one deterministic run repeated 30 times.

(24) $$RMSE=\sqrt{\frac{1}{n}\sum_{i=1}^{n}{\left(y_{i}-{\hat{y}}_{i}\right)}^{2}}$$

(25) $$MAE=\frac{1}{n}\sum_{i=1}^{n}\left|y_{i}-{\hat{y}}_{i}\right|$$

(26) $$MAPE=\frac{1}{n}\sum_{i=1}^{n}\left|\frac{y_{i}-{\hat{y}}_{i}}{y_{i}}\right|\times 100\%$$

### 3.3. Analysis of the Impact of Training-Set Size on Model Performance

**Figure 4:**
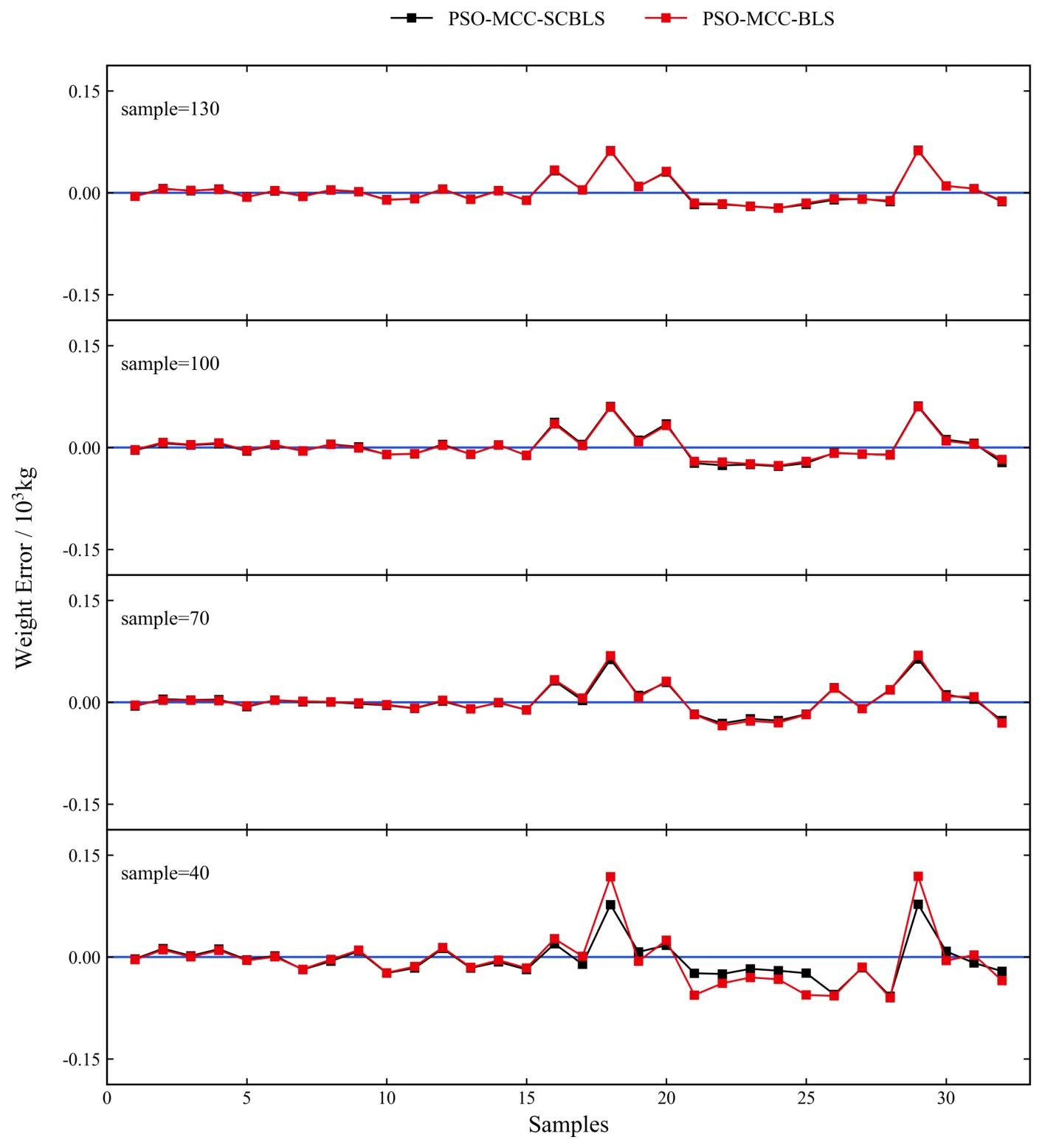
Comparison of weight errors between PSO-MCC-SCBLS and PSO-MCC-BLS under different numbers of training samples. Out-of-fold predictions on fold 5 of the group-wise five-fold split, with the blue horizontal line marking zero error. The 32 test samples are plotted in acquisition order; this fold contains 6 samples at a nominal load of 0.5 t, 12 at 1 t, 8 at 6 t and 6 at 10 t. Because the group-wise split assigns whole calibration groups to folds, the four load levels are neither evenly distributed nor contiguous in the sample index, which is why the error traces do not separate into four equal bands.

To assess the impact of training-data size on model performance, the training set was divided into four subsets of 130, 100, 70, and 40 samples, while the test set remained constant for fair comparison. Subsets are drawn by whole calibration group so as not to split a repeated-measurement pair, so the realized size can fall a few samples short of the target (the nominal-130 subset realizes 128 samples on one of the five folds and exactly 130 on the other four). The PSO-MCC-SCBLS and PSO-MCC-BLS models were trained on each subset for error compensation; the two models differ only in whether the smoothing constraint is active, so the difference between them isolates its contribution. Both were evaluated under group-wise five-fold cross-validation with 30 independently seeded repetitions; each entry is the mean over folds of the per-fold repetition mean, and the reported standard deviation is taken across the five folds. Quantitative error metrics are summarized in Table 1, and Figure 4 visualizes partial-sample prediction residuals, defined as the difference between the ground truth and model output.

Table 1 and Figure 4 show that the benefit of the smoothing constraint is concentrated in the small-sample regime. At the smallest training set (n=40) PSO-MCC-SCBLS reduces RMSE by 13.6% relative to PSO-MCC-BLS (0.0541 against 0.0626), reduces MAE by 10.9%, and produces a visibly smoother error curve in Figure 4; at the three larger sizes the two models are close, with PSO-MCC-BLS nominally ahead. None of the per-size differences is statistically significant: with five folds as the pairing unit, the smallest attainable two-sided Wilcoxon signed-rank *p*-value is $2^{-4}=0.0625$, so no cell of Table 1 could reach the 0.05 level regardless of effect size, and the observed values are p=0.81, 0.63, 0.31 and 0.63 for n=130, 100, 70 and 40. The differences at n≥70 are, therefore, of the same order as the fold-to-fold evaluation noise for a dataset of this size, and only the direction of the effect at n=40 should be read as informative.

Both models degrade as the training set shrinks, but the degradation is far from uniform: between n=130 and n=70 the curves are nearly flat, and only at n=40 does RMSE rise sharply, roughly doubling for both models. This reflects the structure of the dataset rather than a property of the models—the 162 samples contain only 81 distinct calibration groups, so removing groups costs little accuracy until the load–position grid is no longer covered, at which point the loss is abrupt. It is precisely in this regime, where the training data no longer span the operating envelope, that a physically motivated prior has something to add, which is consistent with the pattern in Table 1. These results support the use of the smoothness constraint for limited-data scenarios, while indicating that it neither helps nor harms materially once the calibration grid is adequately covered.

### 3.4. Model Validation and Analysis

To determine which components of the proposed system are actually responsible for its performance, PSO-MCC-SCBLS was compared under normal weighing conditions against the six reduced variants of the BLS family obtained by switching each component on or off—BLS, MCC-BLS, SCBLS, PSO-BLS, PSO-SCBLS and PSO-MCC-BLS—and against RBLS, CatBoost, CNN_Attention, and LSTM. All models underwent group-wise five-fold cross-validation with independent repetitions—30 for the BLS-family variants and 3 per fold for the four external baselines, which are not re-tuned per fold—to assess prediction accuracy and stability. The naming follows the components each variant carries: the PSO- prefix marks automated structural search, MCC marks the maximum correntropy criterion in place of MMSE, and SC marks the smoothness constraint, so that BLS is the plain baseline and PSO-MCC-SCBLS the full model. An uncompensated reference, obtained by summing the eight raw channel outputs without any zero-offset removal or span calibration, is included solely to indicate the magnitude of the raw error.

The parameter settings for the BLS-family variants are aligned with those of PSO-MCC-SCBLS; the three variants without the PSO- prefix are tuned by a random grid search of the same evaluation budget, so that the effect of the search strategy is not confounded with the size of the tuning budget. The RBLS model employs a random mapping structure akin to that of the standard BLS, utilizing 10 feature windows, 10 feature nodes per window, and 100 enhancement nodes. To maintain mathematical stability and noise robustness in small-sample scenarios, the output weights are determined using the Huber robust regression algorithm, with an L2 regularization coefficient of 0.1. The CatBoost model imposes strict limitations on tree complexity to mitigate overfitting in small samples, setting the maximum tree depth to 5, using 400 boosting trees, and a learning rate of 0.05. The Bernoulli random subsampling mechanism is activated, with both feature and sample sampling rates set at 0.8. For the deep-learning baselines, LSTM and CNN_Attention, the performance is evaluated using the noise-robust Smooth L1 loss function rather than the standard MSE. Both models utilize the Adam optimizer with weight decay, a batch size of 16 based on the sample size, and a maximum of 300 training epochs. Specifically, the LSTM architecture consists of two hidden layers, features a hidden state dimension of 32, and incorporates a dropout rate of 0.2. The CNN_Attention network comprises a 1D convolutional layer featuring 16 channels, a Squeeze-and-Excitation (SE) channel attention module, and a fully connected regression layer with 32 neurons. To validate the importance of performance distinctions among models, the Friedman test is employed to statistically assess the RMSE values of the prediction outcomes from each model.

Because the structural search is repeated independently on every training fold, the PSO-tuned variants do not have a single global parameter setting. Across the five folds and 30 repetitions the median selected configuration of PSO-MCC-SCBLS is n=1 feature window, k=9 feature nodes per window and m=2 enhancement nodes, with interquartile ranges of n∈[1,6] and k∈[2,9]; for PSO-SCBLS the medians are n=8, k=2 and m=2. The search drives the number of enhancement nodes to the lower bound of the interval in every fold, that is, PSO restricts the nonlinear capacity of the network rather than expanding it. On a dataset of 162 samples containing only 81 distinct calibration groups, this is the behavior that a correctly regularized structural search should exhibit, and it is the first indication that the clean-data problem is close to a low-complexity regime.

Table 2 presents the predictive results of each model under group-wise five-fold cross-validation, and Table 3 the corresponding Friedman rank test. Compensation is indispensable in absolute terms: the uncompensated eight-channel sum has an RMSE of 9.0936, so every BLS-family variant removes more than 98.7% of the raw error. Among the calibrated models, however, the corrected evaluation protocol gives a picture that is markedly less favorable to the full model than the original submission. The lowest RMSE is obtained by PSO-MCC-BLS (0.0209) and PSO-BLS (0.0219), while the full PSO-MCC-SCBLS model records 0.0292 and PSO-SCBLS 0.0297. We report this directly rather than concealing it: on clean data the results do not support an accuracy claim for the full model. What they do show is that once repeated-measurement groups are kept on the same side of the train/test split and the hyperparameters are selected inside the training folds only, the truck-scale calibration problem is already close to a low-complexity, near-linear regime, in which the additional capacity contributed by the smoothness constraint has nothing left to explain and acts only as an extra source of variance—visible in the standard deviation of PSO-MCC-SCBLS (0.0200), which is six times that of PSO-MCC-BLS (0.0031).

The component-level reading of Table 2 is, nevertheless, informative. Automated structural search is the one component whose benefit is unambiguous and large: PSO-BLS improves on plain BLS by 47.7% in RMSE (0.0419 to 0.0219) and PSO-MCC-BLS improves on MCC-BLS by 51.7% (0.0433 to 0.0209), and the same ordering holds for MAE and MAPE. The other two components do not survive the corrected protocol as independent contributors. The MCC criterion changes RMSE by −3.3% without PSO (BLS 0.0419 against MCC-BLS 0.0433) and by +4.6% with it (PSO-BLS 0.0219 against PSO-MCC-BLS 0.0209), that is, it moves the result by less than the between-fold standard deviation in either direction. The smoothness constraint is the clearest negative: without PSO it degrades RMSE almost threefold (BLS 0.0419 against SCBLS 0.1151), and with PSO it degrades it by 35.6% (PSO-BLS 0.0219 against PSO-SCBLS 0.0297). Its usefulness is confined to the small-sample regime documented in Section 3.3 and to the disturbed-deployment condition of Section 3.5.

**Figure 5:**
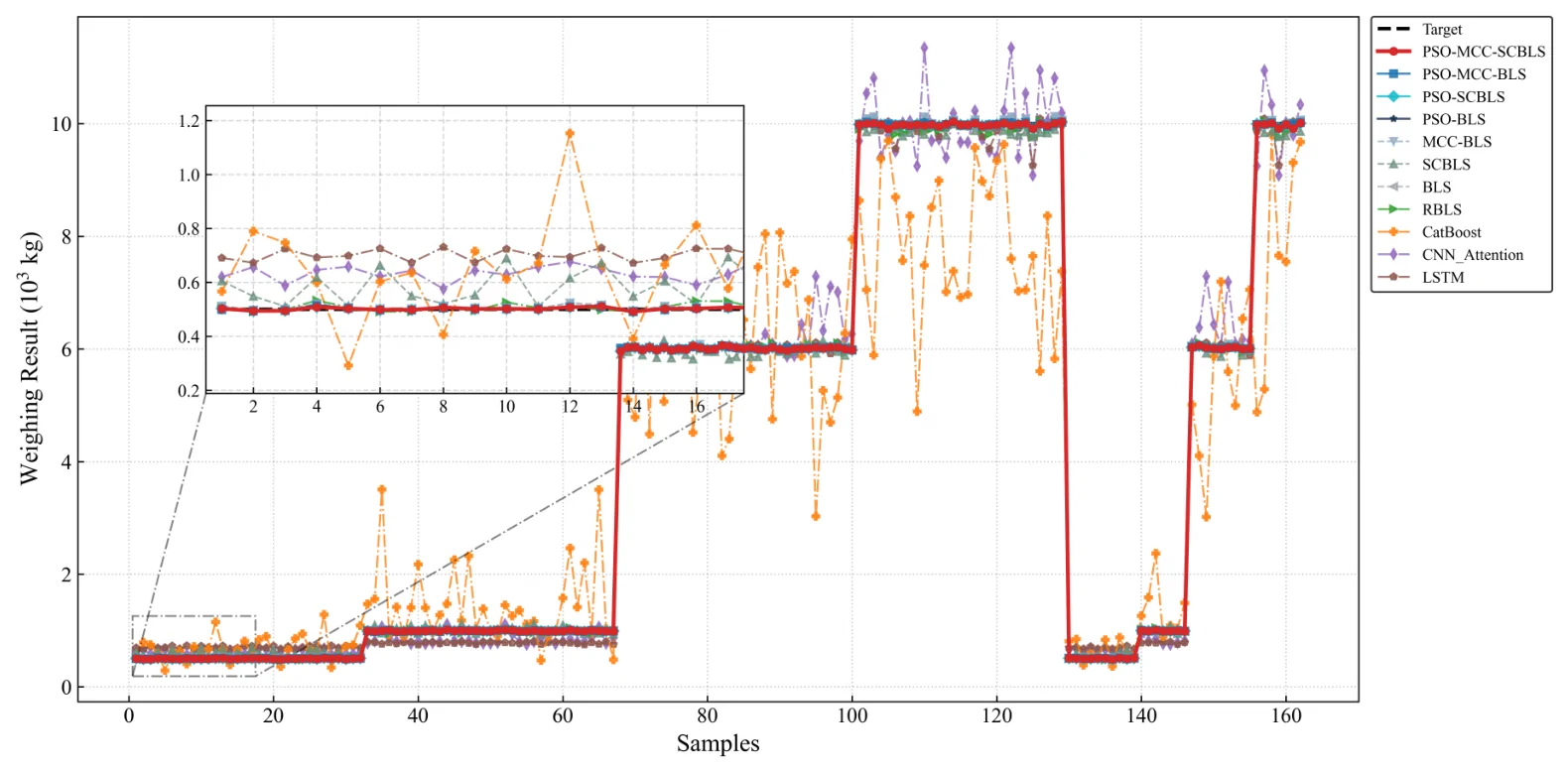
Predicted output comparison of each model under normal conditions. All eleven compensation models from Table 2 are plotted; the inset magnifies the 0.5–1 t range, where the four PSO-tuned variants overlap the target curve.

**Figure 6:**
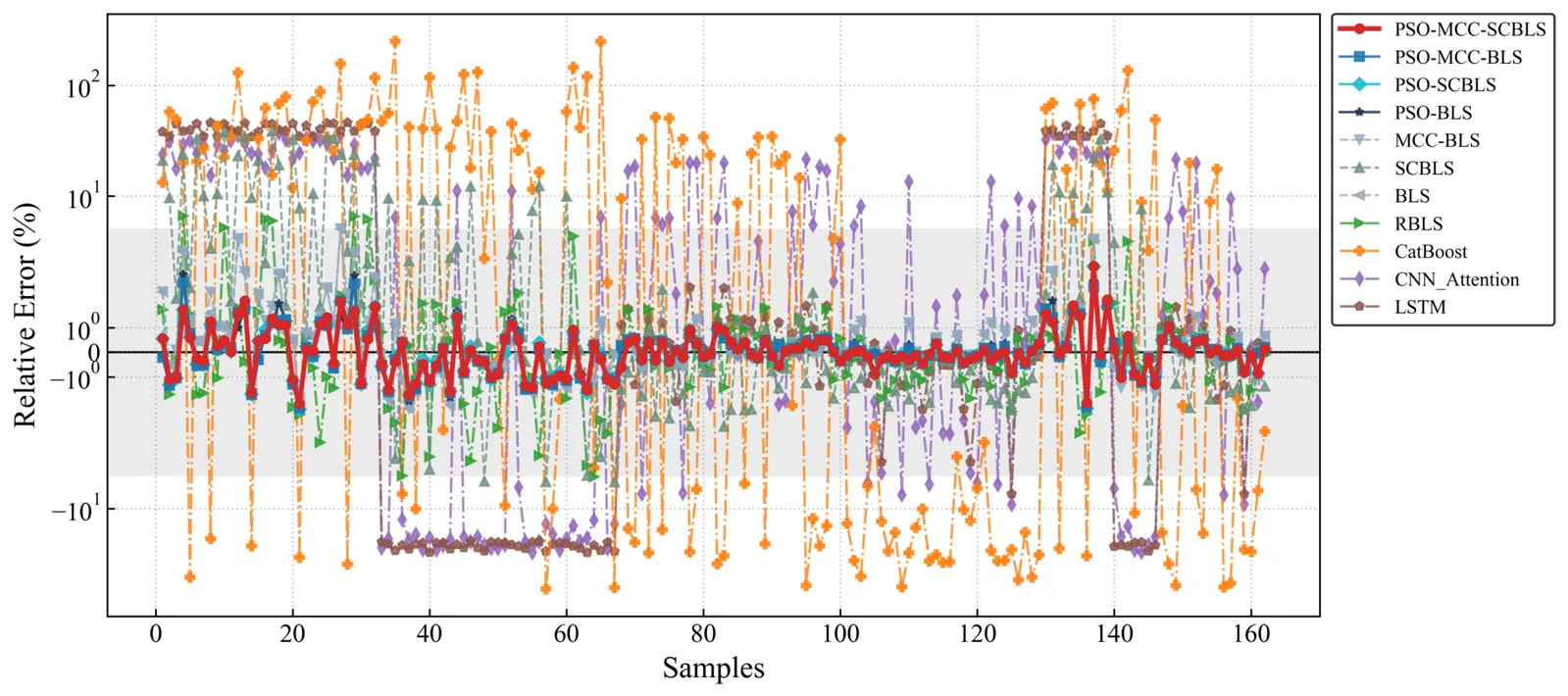
Relative error comparison of each model under normal conditions. The vertical axis is symmetric-logarithmic, with the shaded band marking the linear region (±5%); this shows every curve in full, including the CatBoost excursions beyond 200%, without compressing the near-zero traces of the BLS-family variants. Relative error is amplified at the 0.5 t load, the lightest in the dataset.

**Figure 7:**
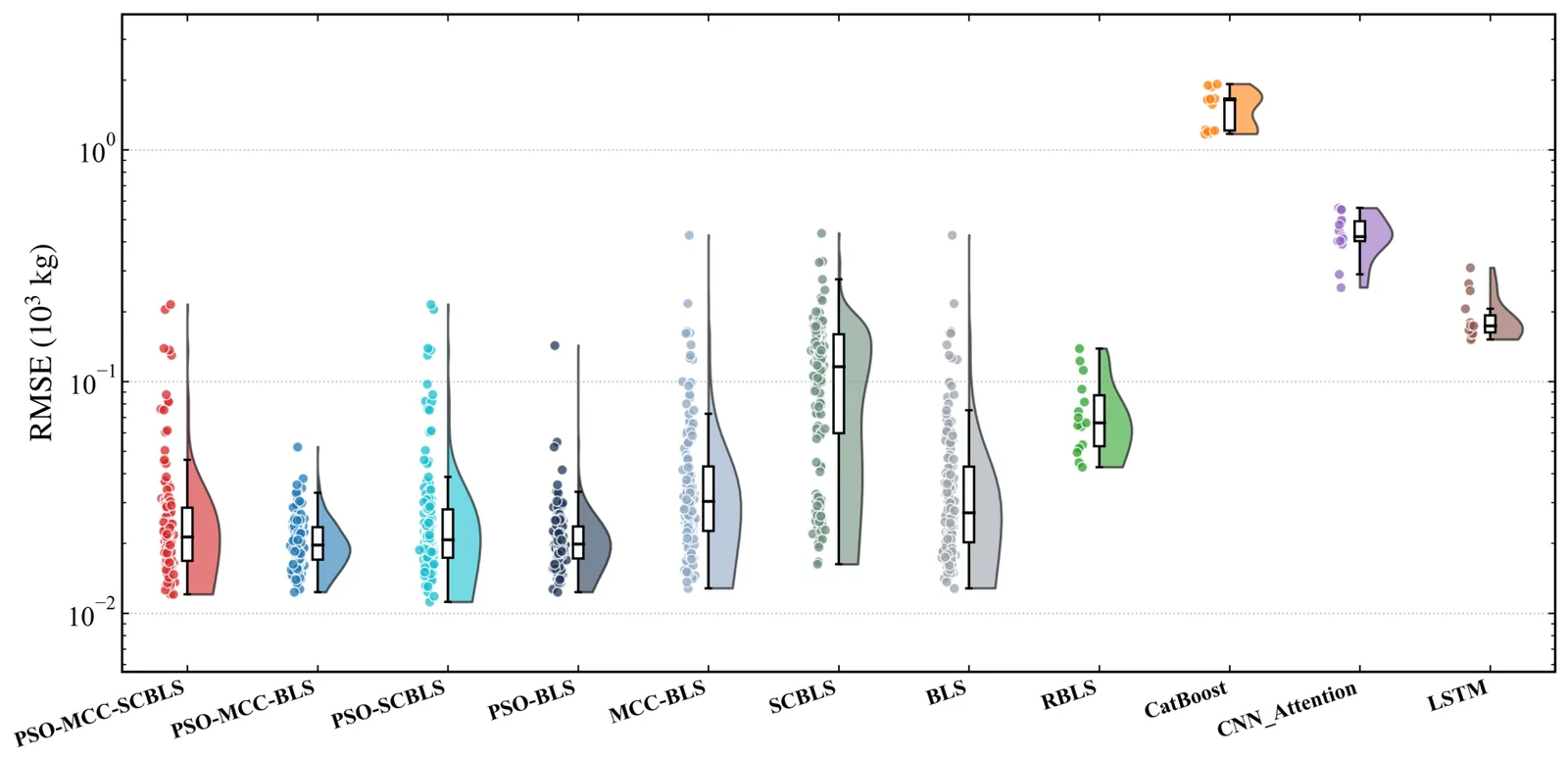
RMSE raincloud plot of each model under normal conditions. Each raincloud shows the per-fold, per-repetition test RMSE as jittered points, a half violin for the density and a mini box plot for the quartiles. The vertical axis is logarithmic because CatBoost exceeds the leading BLS-family variants by almost two orders of magnitude and a linear axis would collapse them onto a single line.

The results of the Friedman test are summarized in Table 3. The test over the eleven compensation models is highly significant ($\chi^{2}=43.78$, $p=3.60\times 10^{-6}$), but the rank ordering separates the over-flexible baselines from the near-linear BLS variants rather than isolating a single winner: the four PSO-tuned variants occupy the first four ranks with mean ranks between 2.40 and 3.40, a spread far smaller than the gap to the next group. This is consistent with the RMSE reading above and does not justify treating MCC or the smoothing constraint as independently beneficial on clean data. To provide a more intuitive comparison of the compensation effects under different loads, Figure 5 displays the predicted output curves of each model in a normal environment. The four PSO-tuned variants track the target values closely across the whole load range and are visually indistinguishable from one another at this scale, while CatBoost, CNN_Attention and LSTM show the pronounced fluctuations expected of over-parameterized models on 130 training samples. Figure 6 shows the corresponding relative errors, and Figure 7 the distribution of the test RMSE of each model.

### 3.5. Verification and Analysis of Model Anti-Interference Ability

To enhance robustness against real-world sensor fluctuations, noise proportional to each feature’s standard deviation was added to the sensor data. This adaptive noise injection accounts for varying sensor scales and fluctuations, preventing feature imbalance from uniform noise. Unlike the original submission, which injected purely Gaussian white noise, the disturbance used here is a composite of two components, both of which have a direct physical counterpart in static weighing: a Gaussian component $g_{j}$, representing the sensor noise floor and the A/D quantization noise, and an impulsive component $u_{j}$, representing vehicle impact, contactor or inverter switching and A/D glitches. Purely Gaussian noise cannot reproduce the sparse, large-amplitude excursions that dominate an industrial installation, and it is precisely these excursions that the maximum correntropy criterion is designed to suppress. For the *j*-th sensor feature with standard deviation $\sigma_{j}$, the composite disturbance is

(27) $$e_{j}=g_{j}+u_{j},\;g_{j}∼N\;\left(0,{\left(\alpha \sigma_{j}\right)}^{2}\right),\;u_{j}=b_{j}\;s_{j}\;\kappa \;\alpha \;\sigma_{j}$$

where α is the noise ratio coefficient, $b_{j}∼\mathrm{Bernoulli}\left(p\right)$ selects independently for each entry whether it receives an impulse (p=0.05), $s_{j}$ is an independent random sign drawn uniformly from {−1,+1}, and κ=3 sets the impulse amplitude relative to the scale $\alpha \sigma_{j}$ of the Gaussian component. The two components are independent, so a given entry may, in principle, receive both. Periodic noise is deliberately excluded: the 162 calibration samples are independent stable readings rather than a time series, so treating the sample index as a time axis would inject an artificial row-order signal rather than a physical disturbance. Consequently, the corrupted sample can be represented as follows:

(28) $$x_{j}^{\prime }=x_{j}+e_{j}$$

**Figure 8:**
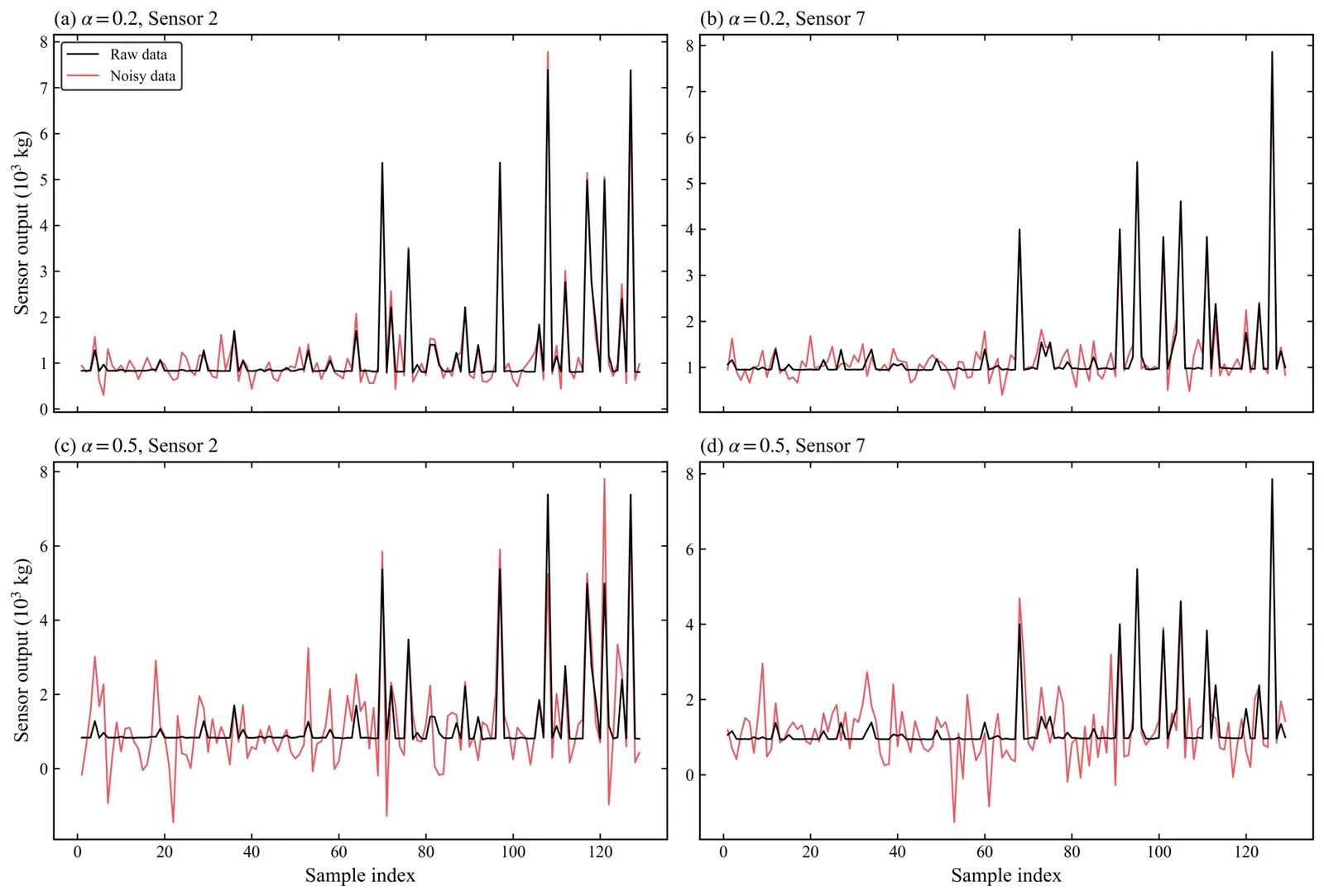
Comparison between original data and noisy data for selected sensors: (**a**) α=0.2, training data of Sensor 2; (**b**) α=0.2, training data of Sensor 7; (**c**) α=0.5, training data of Sensor 2; and (**d**) α=0.5, training data of Sensor 7. The sparse tall spikes are the impulsive component of Equation (27).

Noise is injected under three scenarios, distinguished by which stage of the workflow is corrupted: (A) only the training (calibration) data, as in the original submission, which corresponds to a disturbed calibration campaign followed by acceptance testing in a quiet environment; (B) both the training and the test (deployment) data, which corresponds to an instrument that permanently operates in a disturbed installation; and (C) only the test (deployment) data, that is, clean calibration followed by deployment in a disturbed environment. Scenario B is the operating condition this method is intended for and is, therefore, the primary setting reported below, with Scenario C as its mirror-image stress test; Scenario A is summarized only briefly, since it defines a boundary rather than an operating point. Table 4 details the standard deviation of each sensor alongside the Gaussian-component noise intensities applied at α=0.2 and α=0.5; the corresponding impulse amplitude is three times the tabulated value, applied to 5% of the entries. To visualize the impact of the noise, Sensors 2 and 7 were randomly selected; Figure 8 depicts the comparison curves between the original data and the data corrupted by noise at the two intensities. Anti-interference experiments were conducted for the same eleven models compared in Section 3.4, all of which were trained and tested using group-wise five-fold cross-validation. Because a single noise realization per fold yields only five paired units—for which the smallest attainable two-sided Wilcoxon *p*-value is $2^{-4}=0.0625$, so that no result could reach significance regardless of effect size—four independent noise realizations were drawn per fold, giving 20 paired units (fold × realization) per stratum. Individual realizations differ by 5–27% in RMSE, which is of the same order as the effects being compared, so all statistics are reported over the pooled 20-unit strata and no single realization is quoted on its own. The Friedman test is applied within each (scenario, α) stratum over the 20 paired units.

**Scenario A (disturbed calibration, clean deployment).** This scenario is a boundary of applicability rather than an operating point, and is, therefore, reported in compressed form. Injecting noise into the calibration data alone raises the RMSE of every model by roughly an order of magnitude relative to clean data, and it reorders the BLS family in a way that mirrors Section 3.4: the variants without the smoothing constraint lead, with PSO-MCC-BLS at 0.5351±0.1600 (α=0.2) and 0.9887±0.2398 (α=0.5), while the full model follows closely at 0.5486±0.1645 and 1.0108±0.2565. The four PSO-tuned variants occupy the first four Friedman ranks at both intensities (mean ranks 3.95–4.45 at α=0.2 and 4.00–4.70 at α=0.5; $\chi^{2}=97.69$, $p=1.58\times 10^{-16}$ and $\chi^{2}=105.73$, $p=3.87\times 10^{-18}$), ahead of the three untuned BLS variants and all four external baselines. The mechanism is straightforward: the training noise is absorbed into the flexible nonlinear mapping, and a smoothness prior fitted on corrupted inputs propagates that corruption. The practical consequence is a deployment rule—the input distribution at inference must be no cleaner than the one used for calibration—and it is Scenario B that satisfies it.

**Figure 9:**
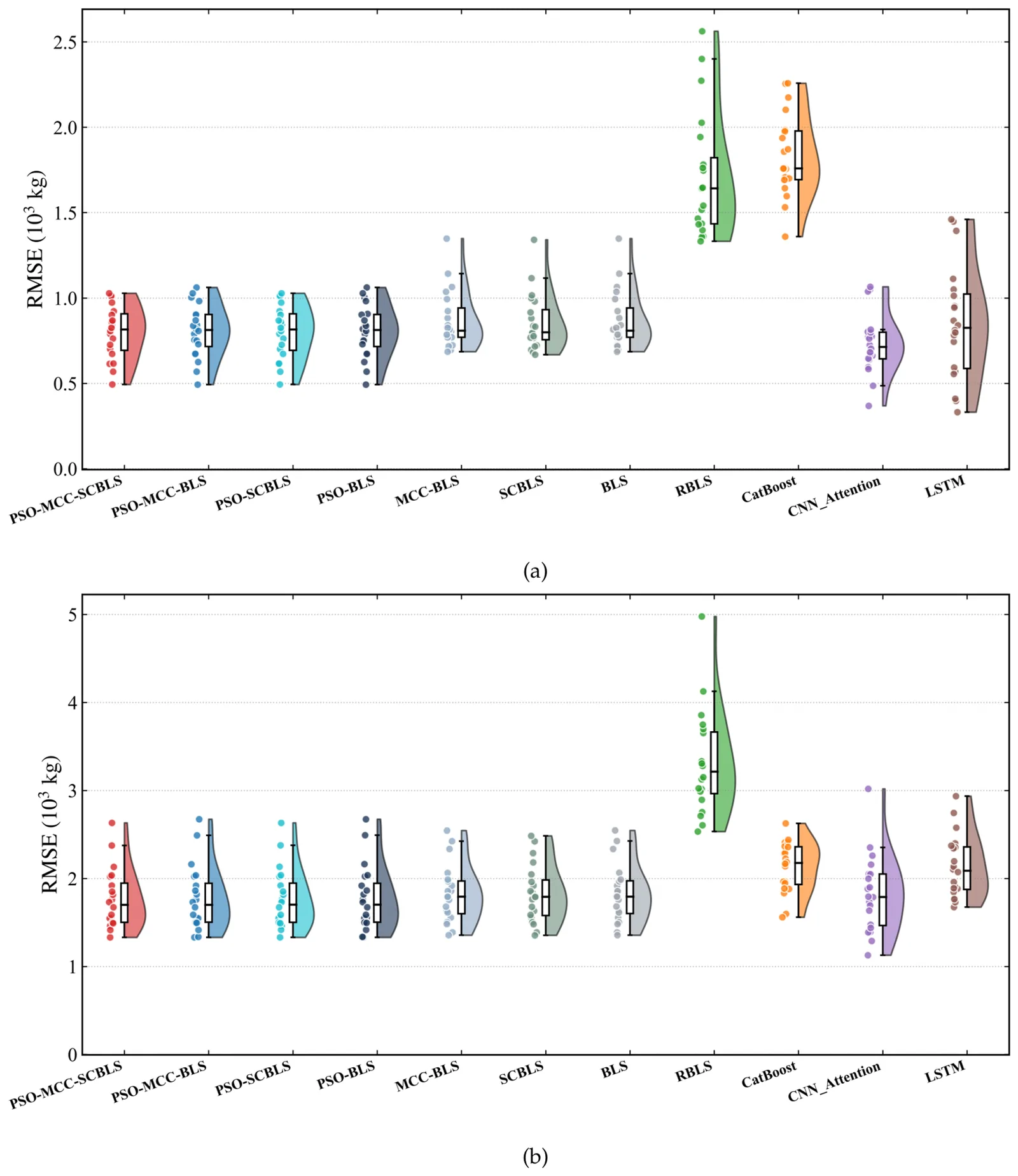
RMSE raincloud plot of each model under Scenario B: (**a**) α=0.2; (**b**) α=0.5. Each raincloud shows the 20 paired units (5 folds × 4 independent noise realizations) as jittered points, a half violin for the density and a mini box plot for the quartiles.

**Figure 10:**
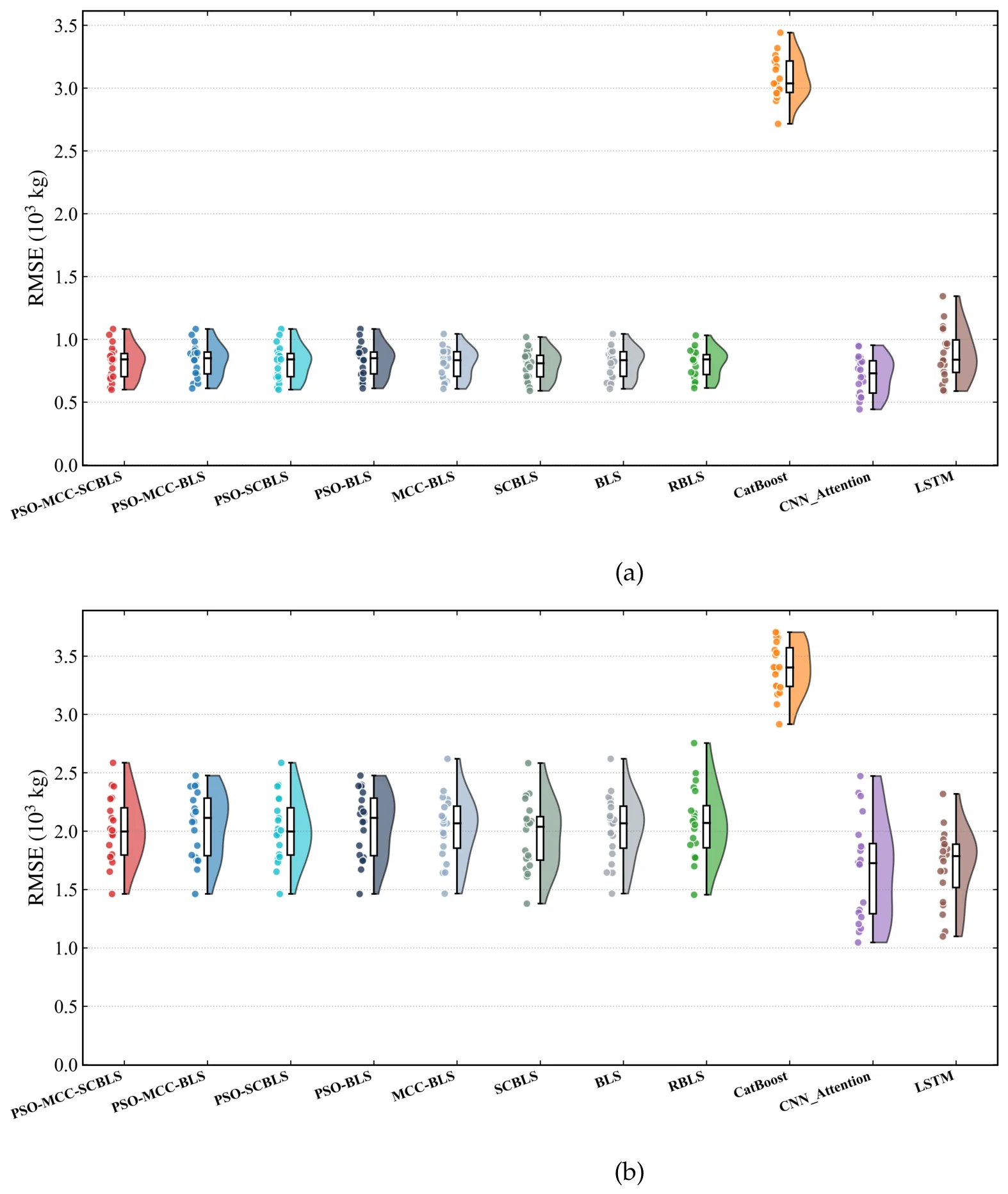
RMSE raincloud plot of each model under Scenario C: (**a**) α=0.2 and (**b**) α=0.5. Plot elements as in Figure 9. Note that the untuned SCBLS variant sits below all four PSO-tuned variants here, the reverse of every other setting.

**Figure 11:**
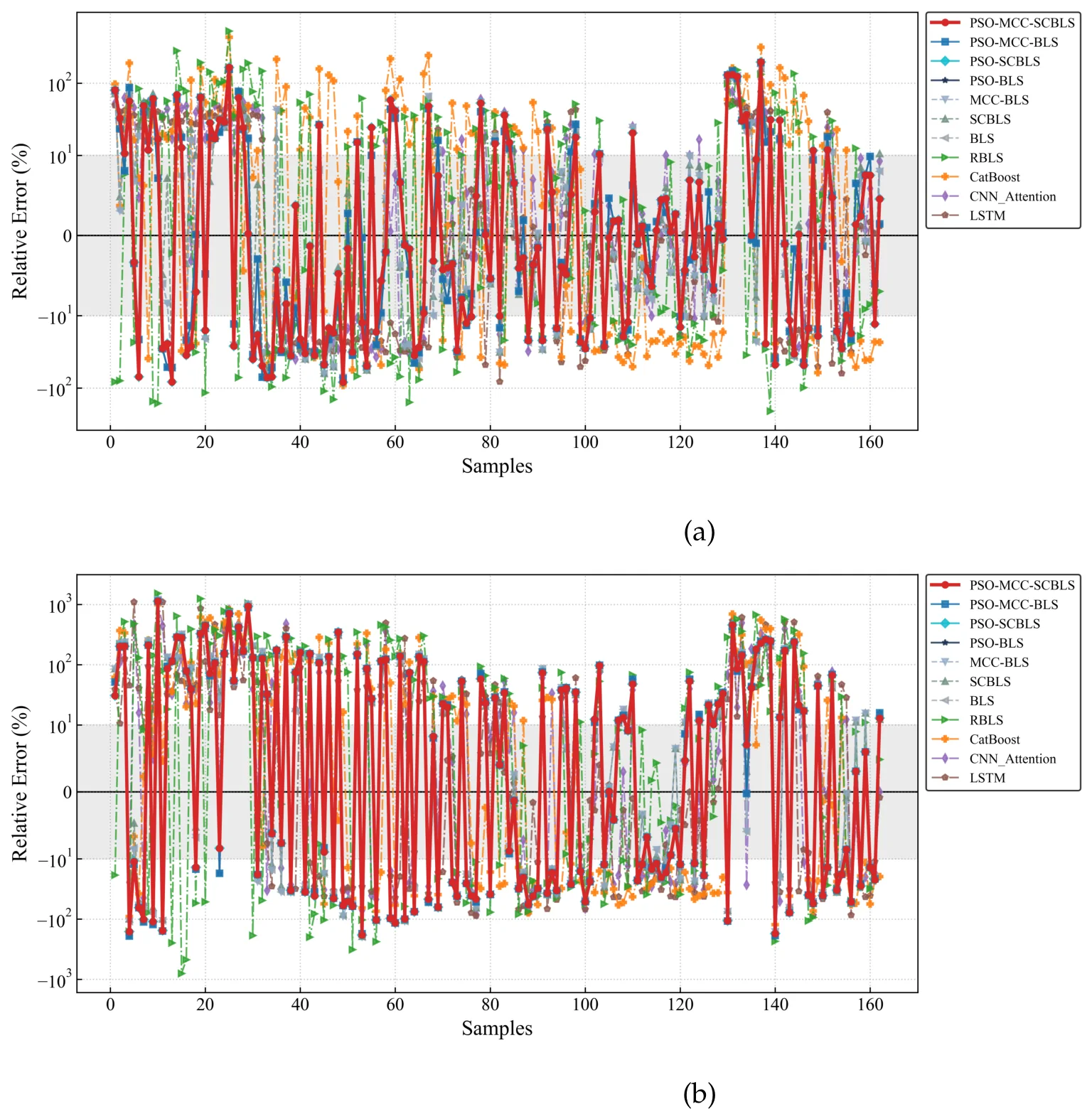
Relative error comparison of each model under Scenario B: (**a**) α=0.2 and (**b**) α=0.5. The vertical axis is symmetric-logarithmic, with the shaded band marking the linear region; this keeps every curve complete while leaving the near-zero traces readable. Relative error is amplified at the 0.5 t load, the lightest in the dataset.

**Scenario B (calibration and deployment both disturbed).** Table 5 and Figure 9, Figure 10 and Figure 11 report this setting, which is the condition the proposed system is designed for. At the stronger intensity α=0.5 the full PSO-MCC-SCBLS model gives the lowest RMSE of all eleven models (1.7670±0.3389) and the best Friedman rank (3.35; $\chi^{2}=91.48$, $p=2.72\times 10^{-15}$), ahead of PSO-MCC-BLS (1.7710), the untuned BLS variants (1.8257–1.8264) and every external baseline, with CNN_Attention at 1.8209, CatBoost at 2.1269, LSTM at 2.1331 and RBLS at 3.3051. This is the one operating point at which the complete model, with all three components active, is the best of the set, and the ordering within the BLS family is the expected one: the smoothing constraint helps (1.7670 against 1.7710 without it), and automated structural search helps more (1.7670 against 1.8264 for untuned SCBLS). At the weaker intensity of α=0.2, the picture is different and is reported as such: CNN_Attention attains both the lowest RMSE (0.7323±0.1760) and the best Friedman rank (3.30), with PSO-MCC-SCBLS second on both counts (0.7998±0.1549, rank 3.70). The advantage of the proposed system is, therefore, specific to strong persistent disturbance, and we claim it no more broadly than that.

**Figure 12:**
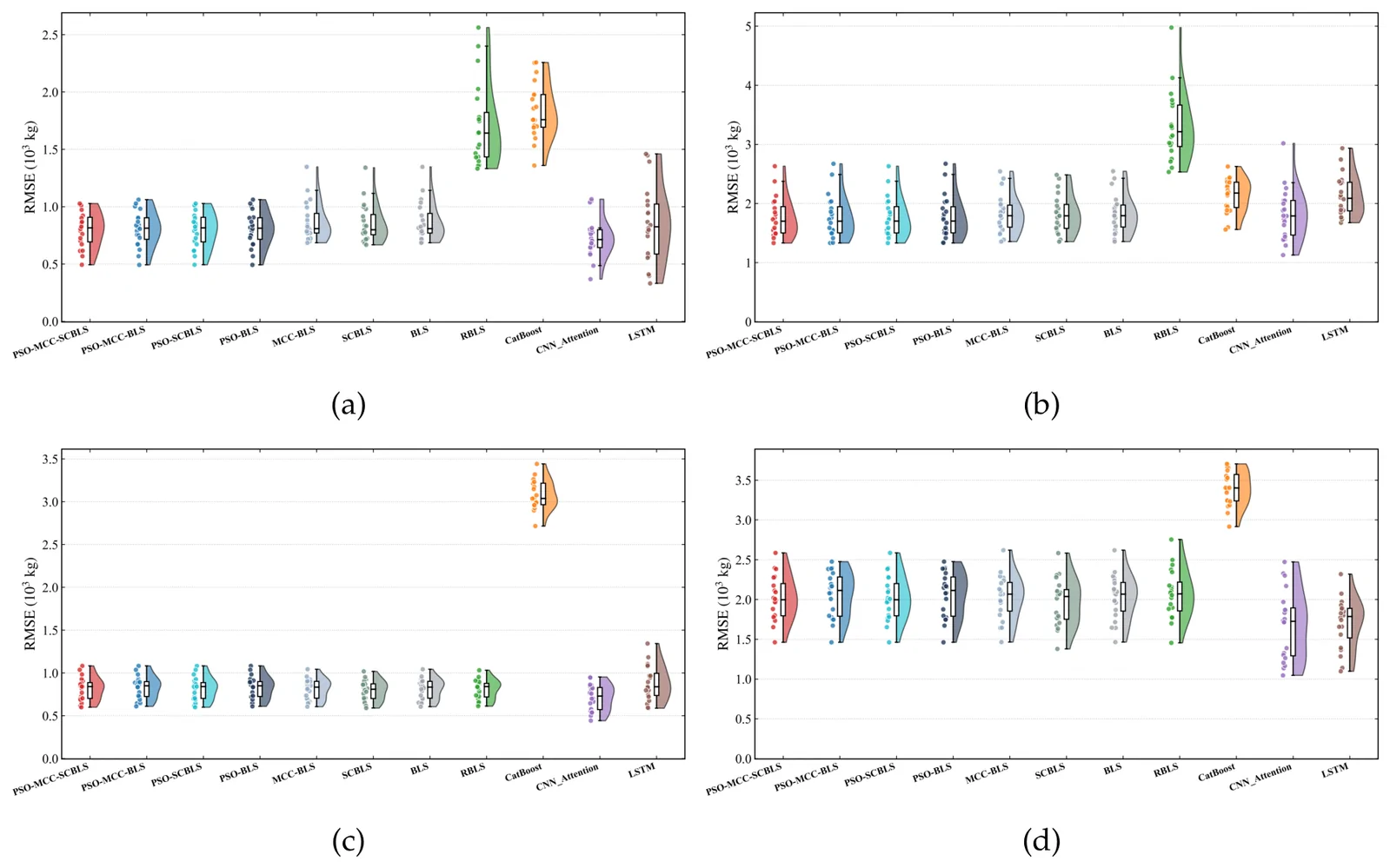
RMSE raincloud plot of each model: (**a**) Scenario B, α=0.2; (**b**) Scenario B, α=0.5; (**c**) Scenario C, α=0.2; and (**d**) Scenario C, α=0.5. Each raincloud shows the 20 paired units (5 folds × 4 independent noise realizations) as jittered points, a half violin for the density and a mini box plot for the quartiles.

**Scenario C (clean calibration, disturbed deployment).** Table 6 and Figure 12 report the mirror image of Scenario A, and it is not a selling point for the proposed method. The two deep-learning baselines lead at both intensities—CNN_Attention at 0.7153±0.1516 (α=0.2) and LSTM at 1.7044±0.3145 (α=0.5)—while PSO-MCC-SCBLS reaches only 0.8165±0.1353 and 2.0166±0.2803, ranking third and fourth, respectively ($\chi^{2}=84.11$, $p=7.82\times 10^{-14}$; $\chi^{2}=129.14$, $p=7.01\times 10^{-23}$). Two observations follow. First, the untuned SCBLS variant outperforms all four PSO-tuned variants in this scenario (0.7942 and 1.9638, Friedman ranks 3.20 and 3.70), which is the reverse of every other setting: a structural search carried out on clean calibration data selects a configuration matched to clean inputs, and that configuration is the wrong one once the deployment inputs are disturbed. Second, this is the only scenario in which the smoothing constraint is clearly and consistently beneficial within each PSO pair, which is what a smoothness prior should do when the disturbance is confined to the inference inputs. Together with Scenario A, this delimits the applicability of the method: it is the matched condition of Scenario B, not disturbance as such, that the proposed system exploits. The corresponding Friedman ranks for Scenarios B and C are summarized in Table 7.

Finally, there is a component-level observation that runs through all three scenarios. Because the effective ridge strength γ is tuned directly rather than through λ (Section 3.2), the MCC and non-MCC variants are compared at matched regularization, and under that comparison the MCC contribution is negligible on this dataset: PSO-MCC-SCBLS and PSO-SCBLS agree to three or four decimal places in every stratum of Table 5 and Table 6, and PSO-MCC-BLS behaves the same as PSO-BLS. This is consistent with the mechanism of Section 2.4—the training residuals remain small relative to the kernel width σ, so the correntropy weights stay close to unity, $\Lambda_{w}$ in Equation (21) is numerically close to the identity and Equation (22) reduces to the ridge solution. The robustness observed in Scenario B should, therefore, be attributed to the complete tuned BLS-family model under matched regularization, and not to MCC as an isolated outlier-suppression component. It also follows that the MCC term would only be expected to act on an installation whose residuals do contain outliers of a magnitude comparable to σ, which this well-conditioned eight-channel scale does not produce.

## 4. Conclusions

To address the error-compensation challenges inherent in truck-scale weighing—arising from coupled nonlinearity, eccentric loading, and environmental noise—this paper proposes an adaptive smoothing-constrained broad learning system (PSO-MCC-SCBLS). The method integrates three architectural components: particle swarm optimization (PSO) to optimize the structural hyperparameters of the BLS; the maximum correntropy criterion (MCC) in place of the conventional minimum mean square error (MMSE) criterion for training the output weights; and a smoothing-constraint mechanism derived from the physical characteristics of the system.

The evaluation protocol has been corrected in four respects that materially affect how the numbers should be read. The 162 samples form 81 groups of repeated measurements, so all cross-validation is group-wise, keeping both replicates of a group on the same side of every split. All structural hyperparameters are selected by an inner three-fold cross-validation computed on the training folds only, so the outer test fold never participates in model selection. The MCC and non-MCC variants are compared at a matched effective regularization strength, since the MCC objective enters the normal equations through $\gamma =\lambda \sigma^{2}$ rather than through λ. Finally, the injected disturbance is a composite of a Gaussian and an impulsive component, applied in three scenarios and evaluated over 20 paired units per stratum.

Under this protocol the contribution of each component can be stated precisely. Automated structural search is the one component whose benefit is unambiguous: it reduces clean-data RMSE by 47.7% relative to a plain BLS (0.0419 to 0.0219) and by 51.7% in the MCC pair (0.0433 to 0.0209), and the PSO-tuned variants occupy the leading Friedman ranks in every scenario except the disturbed-deployment case. On clean data the full model does not lead: PSO-MCC-BLS reaches 0.0209 and PSO-BLS 0.0219, while PSO-MCC-SCBLS records 0.0292, with a six-fold larger between-fold standard deviation. We report this directly. It indicates that, once repeated-measurement groups are kept together and hyperparameters are chosen inside the training folds, the truck-scale calibration problem is already close to a low-complexity regime in which the extra capacity of the smoothing constraint contributes variance rather than accuracy.

The defensible advantage of the full model is narrower and lies where the method was intended to operate. In Scenario B, where both calibration and deployment data are disturbed at α=0.5, PSO-MCC-SCBLS attains the lowest RMSE of all eleven models ($1.7670\times 10^{3}$ kg) and the best Friedman rank (3.35, $p=2.72\times 10^{-15}$), ahead of every ablation variant and every external baseline; the ordering within the BLS family is the expected one, with the smoothing constraint and the structural search each contributing. The boundaries are equally clear and are reported rather than omitted: at α=0.2 in the same scenario, CNN_Attention leads on both RMSE and rank; in Scenario C, where calibration is clean and only the deployment inputs are disturbed, CNN_Attention and LSTM lead and the untuned SCBLS variant outperforms all four PSO-tuned variants because a structure selected on clean data is matched to clean inputs; and Scenario A, where only the calibration data is disturbed, is a boundary rather than an operating point. Matched regularization further shows that MCC provides no measurable isolated contribution to this dataset, since the training residuals of this well-conditioned eight-channel installation remain far below the correntropy kernel width. The practical value of the framework is, therefore, its tuned BLS-family robustness under strong, persistent disturbance that reaches both calibration and deployment, not an across-the-board accuracy improvement.

Three limitations follow directly. The dataset covers 81 distinct load–position combinations on a single 40 t installation, so the reported margins remain to be verified on other scale geometries and capacities. The four external baselines use fixed hyperparameters and are not re-tuned per fold, whereas the BLS-family variants perform a nested structural search on every training fold, so the comparison credits the proposed method with its automated search. And the MCC term should be re-examined on an installation whose residuals do contain outliers comparable to the kernel width, which is the condition under which it is designed to act. Extending the automated search to the deep-learning baselines under an equal tuning budget, and validating the method on additional installations, are the natural directions for future work.

## Figures and Tables

**Table 1:** Statistical comparison of errors for PSO-MCC-SCBLS and PSO-MCC-BLS under varying training set sizes. Bold values indicate the lower mean error for each metric and training-set size.

Model	Evaluation Metrics	Statistics	Training Set Size			
			130	100	70	40
PSO-MCC-SCBLS	RMSE ($10^{3}$ kg)	Mean	0.0292	0.0234	0.0244	**0.0541**
		Std	0.0200	0.0055	0.0028	0.0180
	MAE ($10^{3}$ kg)	Mean	0.0191	0.0170	0.0172	**0.0327**
		Std	0.0107	0.0032	0.0024	0.0087
	MAPE (%)	Mean	0.7439	0.7950	0.8339	**1.1278**
		Std	0.2001	0.1648	0.1999	0.1827
PSO-MCC-BLS	RMSE ($10^{3}$ kg)	Mean	**0.0209**	**0.0214**	**0.0225**	0.0626
		Std	0.0031	0.0052	0.0034	0.0187
	MAE ($10^{3}$ kg)	Mean	**0.0150**	**0.0155**	**0.0162**	0.0367
		Std	0.0018	0.0028	0.0024	0.0090
	MAPE (%)	Mean	**0.7090**	**0.7364**	**0.7820**	1.1917
		Std	0.0899	0.1269	0.1846	0.1849

**Table 2:** Statistical Comparison of Model Errors under Normal Conditions. Values are mean ± standard deviation across the five cross-validation folds, ordered by RMSE; *e* is the folded division value (e=10 kg) and %FS is relative to the 40 t full scale. The uncompensated row is the sum of the eight raw channel readings without zero-offset removal or span calibration, reported only to indicate the magnitude of the uncorrected signal. Bold values indicate the lowest error among the compensated models for the corresponding metric.

Model	RMSE ($10^{3}$ kg)	MAE ($10^{3}$ kg)	MAPE (%)	*e*	%FS
Uncompensated Value	9.0936	9.0918	778.3428	909.4	22.734
PSO-MCC-BLS	0.0209±0.0031	0.0150±0.0018	0.7090±0.0899	2.09	0.052
PSO-BLS	0.0219±0.0032	0.0159±0.0027	0.7390±0.1511	2.19	0.055
PSO-MCC-SCBLS	0.0292±0.0200	0.0191±0.0107	0.7439±0.2001	2.92	0.073
PSO-SCBLS	0.0297±0.0196	0.0196±0.0104	0.7543±0.1990	2.97	0.074
BLS	0.0419±0.0213	0.0265±0.0121	1.0567±0.5472	4.19	0.105
MCC-BLS	0.0433±0.0197	0.0276±0.0109	1.0822±0.5285	4.33	0.108
RBLS	0.0752±0.0234	0.0544±0.0160	2.5218±0.6000	7.52	0.188
SCBLS	0.1151±0.0529	0.0870±0.0492	5.5761±4.6689	11.51	0.288
LSTM	0.1903±0.0474	0.1583±0.0304	16.7119±0.5260	19.03	0.476
CNN_Attention	0.4369±0.0842	0.3086±0.0559	14.6375±0.8467	43.69	1.092
CatBoost	1.5145±0.3081	1.0376±0.1724	36.8102±8.9757	151.45	3.786

**Table 3:** Results of the Friedman test on the RMSE values of the models. Blocks are the five cross-validation folds and a lower mean rank is better; $\chi^{2}=43.78$, $p=3.60\times 10^{-6}$.

Rank	Algorithm	Friedman Test	Rank	Algorithm	Friedman Test
1	PSO-BLS	2.40	7	RBLS	6.80
2	PSO-MCC-BLS	2.60	8	SCBLS	7.60
3	PSO-MCC-SCBLS	3.20	9	LSTM	9.00
4	PSO-SCBLS	3.40	10	CNN_Attention	10.00
5	BLS	4.00	11	CatBoost	11.00
6	MCC-BLS	6.00	*p*-value		<0.05

**Table 4:** Standard Deviation of Different Sensors and the Intensity of the Gaussian Component of the Injected Noise at Various Proportions (Unit: $10^{3}$ kg).

Sensors	Std	α=0.2	α=0.5
Sensor 1	1.0173	0.2035	0.5087
Sensor 2	1.0610	0.2122	0.5305
Sensor 3	1.2705	0.2541	0.6353
Sensor 4	1.3082	0.2616	0.6541
Sensor 5	1.2113	0.2423	0.6057
Sensor 6	1.2356	0.2471	0.6178
Sensor 7	1.0827	0.2165	0.5414
Sensor 8	1.0610	0.2122	0.5305

**Table 5:** Comparison of Error Statistics for Interference Resistance Results of Each Model under Scenario B (both calibration and deployment data disturbed). Values are mean ± standard deviation over the 20 paired units (5 folds × 4 independent noise realizations). Bold values indicate the lowest error for the corresponding metric and noise intensity.

Model	α=0.2			α=0.5		
	RMSE ($10^{3}$kg)	MAE ($10^{3}$kg)	MAPE (%)	RMSE ($10^{3}$kg)	MAE ($10^{3}$kg)	MAPE (%)
PSO-MCC-SCBLS	0.7998±0.1549	0.5690±0.0930	32.8733±8.6893	1.7670±0.3389	1.3140±0.2431	84.3821±23.7677
PSO-SCBLS	0.7998±0.1549	0.5690±0.0930	32.8734±8.6893	1.7675±0.3391	1.3143±0.2433	84.3937±23.7829
PSO-MCC-BLS	0.8059±0.1529	0.5713±0.0876	32.6839±8.3644	1.7710±0.3642	1.3167±0.2563	85.0384±23.6700
PSO-BLS	0.8059±0.1530	0.5713±0.0876	32.6839±8.3641	1.7715±0.3645	1.3170±0.2565	85.0489±23.6851
MCC-BLS	0.8700±0.1696	0.6082±0.0891	31.8996±9.0178	1.8257±0.3295	1.3408±0.2220	83.2727±23.1945
SCBLS	0.8555±0.1671	0.5980±0.0930	31.5765±9.1978	1.8264±0.3274	1.3377±0.2180	82.3548±23.3201
BLS	0.8701±0.1696	0.6082±0.0891	31.8998±9.0176	1.8263±0.3298	1.3411±0.2223	83.2900±23.2100
RBLS	1.7131±0.3599	1.2691±0.2399	75.6083±13.0045	3.3051±0.5839	2.5761±0.4222	186.5735±26.8103
CatBoost	1.8307±0.2406	1.3100±0.1570	54.8603±13.5300	2.1269±0.2880	1.5853±0.2547	96.6504±28.5876
CNN_Attention	0.7323±0.1760	0.5194±0.0970	27.6836±7.5496	1.8209±0.4369	1.2759±0.3044	77.6229±35.0231
LSTM	0.8538±0.3323	0.5055±0.1857	26.0213±7.0639	2.1331±0.3529	1.3682±0.3365	74.5419±45.4759

**Table 6:** Comparison of Error Statistics for Interference Resistance Results of Each Model under Scenario C (clean calibration and disturbed-deployment data). Values are mean ± standard deviation over the 20 paired units (5 folds × 4 independent noise realizations). Bold values indicate the lowest error for the corresponding metric and noise intensity.

Model	α=0.2			α=0.5		
	RMSE ($10^{3}$kg)	MAE ($10^{3}$kg)	MAPE (%)	RMSE ($10^{3}$kg)	MAE ($10^{3}$kg)	MAPE (%)
PSO-MCC-SCBLS	0.8165±0.1353	0.6366±0.1045	49.0420±12.7220	2.0166±0.2803	1.6022±0.2083	135.3432±19.7176
PSO-SCBLS	0.8165±0.1353	0.6366±0.1045	49.0420±12.7220	2.0166±0.2803	1.6022±0.2083	135.3432±19.7176
PSO-MCC-BLS	0.8281±0.1319	0.6454±0.0999	50.4085±12.2971	2.0578±0.2859	1.6329±0.2130	140.6843±18.6946
PSO-BLS	0.8281±0.1319	0.6454±0.0999	50.4085±12.2971	2.0578±0.2859	1.6329±0.2130	140.6843±18.6946
MCC-BLS	0.8124±0.1203	0.6380±0.0979	49.6047±12.6420	2.0268±0.2782	1.6020±0.1991	139.2218±18.0433
SCBLS	0.7942±0.1184	0.6224±0.0994	47.6330±12.3160	1.9638±0.2987	1.5590±0.2077	133.2866±17.7820
BLS	0.8124±0.1203	0.6380±0.0979	49.6047±12.6420	2.0268±0.2782	1.6020±0.1991	139.2218±18.0433
RBLS	0.8100±0.1155	0.6359±0.0909	49.9928±12.6341	2.0668±0.3110	1.6379±0.2186	138.4359±19.5399
CatBoost	3.0803±0.1699	2.6459±0.1667	230.8524±34.0838	3.3975±0.2216	2.9581±0.2312	271.1601±41.7685
CNN_Attention	0.7153±0.1516	0.5295±0.1042	30.0910±8.9246	1.6780±0.4336	1.2485±0.2977	96.6311±24.8662
LSTM	0.8815±0.2038	0.5332±0.1220	24.1220±3.5324	1.7044±0.3145	1.0699±0.1980	52.0978±16.4437

**Table 7:** Friedman Test Results of RMSE Values for Each Model in a Noisy Environment (Scenarios B and C). Blocks are the 20 paired units (5 folds × 4 noise realizations) and a lower mean rank is better. Scenario B: $\chi^{2}=107.17$, $p=1.98\times 10^{-18}$ (α=0.2) and $\chi^{2}=91.48$, $p=2.72\times 10^{-15}$ (α=0.5); Scenario C: $\chi^{2}=84.11$, $p=7.82\times 10^{-14}$ (α=0.2) and $\chi^{2}=129.14$, $p=7.01\times 10^{-23}$ (α=0.5). Equal mean ranks share a rank, and bold values indicate the best rank or test value within the corresponding stratum.

Algorithm	Scenario B				Scenario C			
	α=0.2		α=0.5		α=0.2		α=0.5	
	Test	Rank	Test	Rank	Test	Rank	Test	Rank
PSO-MCC-SCBLS	3.70	2	**3.35**	**1**	4.75	3	5.05	4
PSO-SCBLS	4.35	3	4.40	2	5.35	4	5.95	6
PSO-MCC-BLS	4.95	4	4.40	2	6.65	8	7.50	8
PSO-BLS	5.50	7	5.55	7	7.05	10	8.00	10
MCC-BLS	6.05	8	5.00	5	6.05	5	5.90	5
SCBLS	5.15	5	4.85	4	3.20	2	3.70	3
BLS	6.85	9	6.00	8	6.05	5	6.80	7
RBLS	10.20	10	10.95	11	6.20	7	7.80	9
CatBoost	10.75	11	8.35	10	11.00	11	11.00	11
CNN_Attention	**3.30**	**1**	5.00	5	**2.95**	**1**	2.30	2
LSTM	5.20	6	8.15	9	6.75	9	**2.00**	**1**
*p*-value <0.05 in all four strata								

## Data Availability

The data presented in this study are available on request from the corresponding author. The data are not publicly available due to privacy constraints.
